# Drug-induced hepatic steatosis in absence of severe mitochondrial dysfunction in HepaRG cells: proof of multiple mechanism-based toxicity

**DOI:** 10.1007/s10565-020-09537-1

**Published:** 2020-06-14

**Authors:** Julien Allard, Simon Bucher, Julie Massart, Pierre-Jean Ferron, Dounia Le Guillou, Roxane Loyant, Yoann Daniel, Youenn Launay, Nelly Buron, Karima Begriche, Annie Borgne-Sanchez, Bernard Fromenty

**Affiliations:** 1 INSERM, Univ Rennes, INRAE, Institut NUMECAN (Nutrition Metabolisms and Cancer) UMR_A 1341, UMR_S 1241, F-35000 Rennes, France; 2 HCS Pharma, 250 rue Salvador Allende, 59120 Loos, France; 3 MITOLOGICS S.A.S, Faculté de Médecine, rue du Général Sarrail, 94000 Créteil, France

**Keywords:** Steatosis, Mitochondria, Fatty acid oxidation, Lipogenesis, Very low-density lipoprotein, Endoplasmic reticulum stress

## Abstract

Steatosis is a liver lesion reported with numerous pharmaceuticals. Prior studies showed that severe impairment of mitochondrial fatty acid oxidation (mtFAO) constantly leads to lipid accretion in liver. However, much less is known about the mechanism(s) of drug-induced steatosis in the absence of severe mitochondrial dysfunction, although previous studies suggested the involvement of mild-to-moderate inhibition of mtFAO, increased de novo lipogenesis (DNL), and impairment of very low-density lipoprotein (VLDL) secretion. The objective of our study, mainly carried out in human hepatoma HepaRG cells, was to investigate these 3 mechanisms with 12 drugs able to induce steatosis in human: amiodarone (AMIO, used as positive control), allopurinol (ALLO), d-penicillamine (DPEN), 5-fluorouracil (5FU), indinavir (INDI), indomethacin (INDO), methimazole (METHI), methotrexate (METHO), nifedipine (NIF), rifampicin (RIF), sulindac (SUL), and troglitazone (TRO). Hepatic cells were exposed to drugs for 4 days with concentrations decreasing ATP level by less than 30% as compared to control and not exceeding 100 × *C*_max_. Among the 12 drugs, AMIO, ALLO, 5FU, INDI, INDO, METHO, RIF, SUL, and TRO induced steatosis in HepaRG cells. AMIO, INDO, and RIF decreased mtFAO. AMIO, INDO, and SUL enhanced DNL. ALLO, 5FU, INDI, INDO, SUL, RIF, and TRO impaired VLDL secretion. These seven drugs reduced the mRNA level of genes playing a major role in VLDL assembly and also induced endoplasmic reticulum (ER) stress. Thus, in the absence of severe mitochondrial dysfunction, drug-induced steatosis can be triggered by different mechanisms, although impairment of VLDL secretion seems more frequently involved, possibly as a consequence of ER stress.

**Electronic supplementary material:**

The online version of this article (10.1007/s10565-020-09537-1) contains supplementary material, which is available to authorized users.

## Introduction

Steatosis (also referred to as fatty liver) is a frequent liver lesion reported in different hepatic diseases including alcoholic liver disease, nonalcoholic fatty liver disease (NAFLD), chronic hepatitis C virus infection, and drug-induced liver injury (DILI) (Amacher and Chalasani 2014; Seitz et al. 2018; Allard et al. 2019). Regarding DILI, Biour and collaborators (Biour et al., 2004) reported that 150 out of 1216 hepatotoxic drugs were able to induce steatosis. In rare cases, drug-induced steatosis can be life-threatening as a consequence of severe mitochondrial dysfunction, which can be favored by different concomitant factors such as genetic predispositions (Amacher and Chalasani 2014; Fromenty 2019; Fromenty and Pessayre 1995). Nevertheless, steatosis is usually asymptomatic or mildly symptomatic in the majority of patients. However, it can progress in the long term in some patients to steatohepatitis, which is characterized by necroinflammation, hepatocyte ballooning, and some fibrosis (Seitz et al. 2018; Begriche et al. 2013; Patel and Sanyal 2013). Hence, it is important to better understand the mechanism(s) whereby drugs can induce steatosis in the absence of severe mitochondrial dysfunction. Previous studies suggested the involvement of mild-to-moderate inhibition of mitochondrial fatty acid oxidation (mtFAO), increased de novo lipogenesis (DNL), and impairment of very low-density lipoprotein (VLDL) secretion (Fromenty 2019; Begriche et al. 2011; Lettéron et al. 2003; Tolosa et al. 2016; Grünig et al. 2018; Rooney et al. 2019). Importantly, these mechanisms are not mutually exclusive and some drugs might alter two or three of these metabolic pathways. For instance, the antianginal and antiarrhythmic drug amiodarone (AMIO) and the antibiotic tetracycline were shown to inhibit mtFAO (Fromenty and Pessayre 1995), but these drugs might also favor DNL via the activation of sterol regulatory element binding protein-1 (SREBP1) (Anthérieu et al. 2011; Corton 2019).

The involvement of these different pathways was based on gene (or protein) expression data (Tolosa et al. 2016; Rooney et al. 2019; Moya et al. 2010; Sahini et al. 2014; Huang et al. 2016), which might not directly reflect the exact pathway(s) leading to the accumulation of lipids (mainly triglycerides). Indeed, gene expression can reflect a bona fide compensatory mechanism in response to drug-induced metabolic dysfunction. For instance, AMIO was shown to impair mtFAO by different mechanisms (Fromenty et al. 1990a; Spaniol et al. 2001; Serviddio et al. 2011), while in mouse liver activation of peroxisome proliferator-activated receptor-α (PPARα) with increased expression of several of its target genes was found (McCarthy et al. 2004; Szalowska et al. 2014), which would suggest increased mtFAO.

In the present study, we selected 12 drugs that were previously reported to induce steatosis in patients: AMIO (used as positive control), allopurinol (ALLO), d-penicillamine (DPEN), 5-fluorouracil (5FU), indinavir (INDI), indomethacin (INDO), methimazole (METHI), methotrexate (METHO), nifedipine (NIF), rifampicin (RIF), sulindac (SUL), and troglitazone (TRO) (Fromenty 2019; Biour et al. 2004; Wang et al. 2013). First, we determined whether these compounds could induce steatosis in differentiated human hepatoma HepaRG cells and primary human hepatocytes (PHH). We aimed to induce steatosis in the absence of severe mitochondrial dysfunction, thus drug concentrations were selected based on a slight or moderate loss (< 30%) of cellular ATP. When steatosis was observed, we next determined the mechanism(s) whereby drugs were able to increase cellular triglycerides by investigating mtFAO, DNL, and VLDL secretion. Altogether, our data confirmed that HepaRG cells is a suitable model to investigate drug-induced steatosis. Moreover, our investigations revealed that only a few steatogenic drugs inhibited mtFAO, or enhanced DNL in HepaRG cells. In contrast, impairment of VLDL secretion seemed to be more often involved in drug-induced lipid accumulation when mitochondrial function is not severely impaired.

## Materials and methods

### Chemicals and reagents

AMIO hydrochloride (reference A8423), ALLO (A8003), DPEN (P4875), 5FU (F6627), INDI (Y0000788), INDO (I7378), METHI (M8506), METHO hydrate (M8407), NIF (N7634), RIF (R3501), SUL (S8139), (+)-etomoxir sodium salt hydrate (E1905), and thapsigargin (T9033) were purchased from Sigma Aldrich (Saint-Quentin-Fallavier, France). Dimethyl sulfoxide (DMSO), digitonin, oleic acid, stearic acid, palmitic acid, palmitoyl-CoA, palmitoyl-l-carnitine, octanoyl-l-carnitine, malate (disodium salt), glutamate (monosodium salt), acetic acid, insulin, l-carnitine, fatty acid-free bovine serum albumin (BSA), and Percoll were also bought from Sigma Aldrich. William’s E medium, Dulbecco’s phosphate-buffered saline (PBS) Gibco™, glutamine, penicillin, streptomycin, formaldehyde, Nile red, and Hoechst 33342 dyes were obtained from Thermo Fischer Scientific (Waltham, MA). TRO (A11981) and lomitapide (A12778) were purchased from Adooq BioScience (Irvine, CA). Tunicamycin (10-2111) and tauroursodeoxycholic acid (TUDCA; 10-2782) were purchased from Focus Biomolecules (Plymouth Meeting, PA). Radiolabeled [U-^14^C]palmitic acid and [2-^14^C]acetic acid, and sodium salt were purchased from PerkinElmer (Waltham, MA). Hydrocortisone hemisuccinate was obtained from SERB Laboratories (Paris, France). Fetal bovine serum (FBS) was purchased from Eurobio (Les Ulis, France) and GE Healthcare (Little Chalfont, UK).

### Cell culture and treatments

Native HepaRG cells were cultured as previously described (Gripon et al. 2002). Briefly, HepaRG cells were seeded in 96-well plates at a density of 2.6 × 10^4^ cells/cm^2^ and were first incubated in William’s E medium supplemented with 10% FBS (50% FBS from Eurobio and 50% FBS from GE Healthcare), 100 U/mL penicillin, 100 μg/mL streptomycin, 2 mM glutamine, 5 μg/mL insulin, and 50 μM hydrocortisone hemisuccinate. After 2 weeks, cell differentiation was stimulated by culturing HepaRG cells in the same medium supplemented with 1.7% DMSO for additional 2 weeks. During this 1-month period, the culture medium was renewed three times a week. Three days before drug treatments, FBS and DMSO concentrations were respectively lowered to 2% and 1%, as previously described (Pomponio et al. 2015). HepaRG cells were next cultured in the same FBS/DMSO conditions and incubated for 4 days with each drug at different concentrations. Culture medium was renewed every day and all investigations were performed after 96 h (i.e., 24 h after the fourth treatment). These investigations were mostly performed in 96-well plates except for western blot analysis, for which 6-well plates were used. Experiments were carried out on passages 9 to 16. All drugs (except DPEN), etomoxir, lomitapide, tunicamycin, thapsigargin, and TUDCA were dissolved in DMSO. DPEN was dissolved in water. Notably, the final concentration of DMSO was fixed at 1% for control and treated cells, regardless of the treatment.

For investigations aiming at preventing drug-induced adverse effects with TUDCA, cells were first pretreated with this chemical chaperone (2 or 4 mM) for 2 h. The culture medium was removed and cells were cotreated by TUDCA and ALLO (750 μM), INDO (300 μM), RIF (300 μM), or tunicamycin (10 μM). The protocol of 4-day treatment was then the same as the one previously described. Because data obtained with 2 and 4 mM TUDCA provided similar results, the data were pooled and compared with control cells.

Primary human hepatocytes (PHH) were purchased from Biopredic International (Saint-Grégoire, France). Cells were seeded by the supplier in coated 96-well plates at a density of 5 × 10^4^ cells/well. Upon receipt, culture medium was removed and replaced by William’s E medium supplemented as previously described for HepaRG cell differentiation, except for DMSO set at 1%. After 2–3 days, cells were treated as for HepaRG cells. For HepaRG cells and PHH, cells were always maintained in incubators at 37 °C with 5% CO_2_ and saturating humidity.

### Selection of drug concentrations

For AMIO, ALLO, 5FU, INDI, INDO, METHO, SUL, RIF, and TRO, hepatic cells were exposed to drug concentrations inducing decrease in ATP level by less than 30% as compared to control (Supplementary Fig. 1). For DPEN, METHI, and NIF, the maximum concentration corresponded to 100 × *C*_max_ (maximum plasma concentrations in patients) (Supplementary Table 1) since there was only slight or no reduction of cellular ATP below this threshold (Supplementary Fig. 1). For INDI, the maximum concentration was 200 μM because of its insolubility for higher concentrations. Hence, for 9 out of the 12 drugs, the respective maximum concentration was below 100 × *C*_max_ (Supplementary Table 1). The 100 × *C*_max_ threshold is classically used in toxicological studies pertaining to DILI (Porceddu et al. 2012; Xu et al. 2008). Therapeutic blood (or plasma) concentrations of these drugs are also provided in Supplementary Table 2.

### Measurement of cellular ATP level

Cellular ATP level was measured using the CellTiter-Glo^®^ Luminescent Cell Viability assay purchased from Promega (Charbonnières, France), according to the manufacturer’s instructions. Briefly, control and treated HepaRG cells were first washed with warm PBS and kept for 30 min at room temperature in phenol red-free William’s E medium. Cells were next incubated with the CellTiter-Glo^®^ reagent for 10 min at room temperature. Cells were then transferred in opaque-walled multiwell plates and the luminescent signal was quantified using a POLARstar Omega microplate reader (BMG Labtech, Ortenberg, Germany). Results were expressed in comparison to control cells.

### Assessment of neutral lipids with Nile red

The fluorescent Nile red dye allows the staining of neutral lipids, namely triglycerides and cholesteryl esters (Greenspan et al. 1985). Nile red staining and neutral lipid quantification were performed as recently described (Bucher et al. 2018), with minor modifications. Briefly, cells were washed with PBS, fixed and stained with PBS containing 4% formaldehyde and 10 μg/mL Hoechst 33342 dye for 30 min, and washed three times with PBS. Cells were then incubated with PBS containing 0.1 μg/mL Nile red for 30 min and washed once. Image acquisition was made with an automated epifluorescence microscope (ImageXpress Micro XLS; Molecular Devices, San Jose, CA) using appropriate excitation/emission wavelengths, namely 350/461 nm for Hoechst 33342 and 531/593 nm for Nile red. For each well, four pictures were taken at a magnification of × 20 and analyzed with Columbus software (PerkinElmer) for quantification. These pictures were always taken in four different areas around the center of each well, and these areas were the same from one well to another, as programmed by the Columbus software. This software was also designed to count only living cells after exclusion of dying cells harboring nuclei with abnormal shape or altered Hoechst intensity. Hence, cell viability was determined by cell counting from Hoechst staining and expressed as a percentage of cells compared to vehicle control condition (Ferron et al. 2014). Neutral lipids were then normalized per number of nuclei and expressed relative to control cells. Examples of images used for neutral lipid quantification are shown in Supplementary Fig. 2.

### Assessment of mtFAO with [U-^14^C]palmitic acid

mtFAO was assessed by measuring the acid-soluble radiolabeled metabolites resulting from the mitochondrial oxidation of [U-^14^C]palmitic acid as previously described (Anthérieu et al. 2011), with slight modifications. Briefly, culture medium was removed and cells were washed with warm PBS before addition of phenol red-free William’s E medium containing 1% fatty acid-free BSA, [U-^14^C]palmitic acid (185 Bq per well, corresponding to 0.5 pM), 100 μM cold palmitic acid, 1 mM l-carnitine, and 1% DMSO. After 3 h of incubation at 37 °C, perchloric acid (final concentration, 6%) was added and plates were centrifuged at 2000×*g* for 10 min, and supernatant was counted for [^14^C]-labeled acid-soluble β-oxidation products using a Tri-Carb 4910TR liquid scintillation counter (PerkinElmer). Results were normalized to total protein content determined using the Pierce BCA assay kit from Thermo Fischer Scientific (Waltham, MA). Results were expressed in comparison to control cells.

### Measurement of oxygen consumption in permeabilized HepaRG cells and isolated mouse liver mitochondria

Cryopreserved differentiated HepaRG cells (HPR116, Biopredic International) were thawed and seeded at 36,000 cells/well in Seahorse XFe96 cell culture microplate (Agilent) in HepaRG^®^ Thawing/Plating/General Purpose Medium Supplement with antibiotics (Biopredic International) combined with 100 mL of William’s Medium E (Sigma) and incubated at 37 °C with 5% CO_2_. The day after, medium was replaced by HepaRG Maintenance/Metabolism Medium Supplement with antibiotics (Biopredic International) combined with 100 mL of William’s Medium E. Seven days post-thawing, cells were permeabilized with digitonin (200 μg/mL) and treated with different concentrations of INDO and RIF. For AMIO, cells were pretreated during 1 h with different concentrations of this drug before digitonin treatment because of an apparent interaction between both compounds. Rapidly after cell permeabilization, oxygen consumption was assessed using a Seahorse XFe96 analyzer (Agilent) in presence of 1 mM malate + 10 μM palmitoyl-CoA + 10 μM l-carnitine, 1 mM malate + 10 μM palmitoyl-l-carnitine, or 1 mM malate + 20 μM octanoyl-l-carnitine. Oxygen consumption was also measured with 1 mM malate + 12.5 mM glutamate, which give their electrons to complex I of the respiratory chain. Measurements were performed for all substrates in presence of 1.65 mM ADP (state 3 respiration). Rotenone (2 μM) was added after 30 min. Two technical replicates were carried out per condition in two to three independent experimental runs. The effective concentration inducing 20% of the maximal effect (EC_20_) assessed for mitochondrial respiration was the drug concentration causing 20% of the maximal inhibition of oxygen consumption achieved with 2 μM rotenone, a prototypical inhibitor of complex I (Buron et al. 2017; Porceddu et al. 2012). EC_20_ calculations, performed using a non-linear regression in GraphPad Prism software V4 (San Diego, CA), were done by compiling the percentage of inhibition obtained from each experiment.

Oxygen consumption of isolated mouse liver mitochondria (incubated or not with different concentrations of RIF) was monitored by spectrofluorimetry, as previously described (Buron et al. 2017; Porceddu et al. 2012). Briefly, liver mitochondria from 6-week-old BALB/cByJ female mice (Charles River, Saint-Germain-sur-L’arbresle, France) were isolated and purified by isopycnic density-gradient centrifugation in Percoll, as previously described (Lecoeur et al. 2004), thus allowing pure and stable mitochondrial preparations (Buron et al. 2017). Isolated mitochondria were then incubated in buffer containing 250 mM sucrose, 30 mM K_2_HPO_4_, 1 mM EGTA, 5 mM MgCl_2_, 15 mM KCl, and 1 mg/mL bovine serum albumin (BSA) supplemented with respiratory substrates and MitoXpress Xtra, an oxygen-sensitive phosphorescent dye purchased from Agilent (Santa Clara, CA). Mitochondrial respiration was measured in presence of 1 mM malate + 12.5 mM glutamate, 1 mM malate + 10 μM palmitoyl-CoA + 10 μM l-carnitine, 1 mM malate + 10 μM palmitoyl-l-carnitine, or 1 mM malate + 20 μM octanoyl-l-carnitine (Buron et al. 2017; Massart et al. 2013; Porceddu et al. 2012). Measurements were performed for all substrates in presence of 1.65 mM ADP (state 3 respiration). For all aforementioned substrates, measurements were performed in parallel without or with 2 μM rotenone in order to calculate (EC_20_), as previously mentioned. Oxygen consumption was measured in real-time for 60 min at 37 °C in 96-well plates using a spectrofluorometer (Tecan Infinite^®^ 200; *λ*_Excitation_ 380 nm; *λ*_Emission_ 650 nm). The slope of fluorescence increase, corresponding to the rate of oxygen consumption, was used for calculations. EC_20_ was thus assessed as previously described by compiling the percentage of inhibition obtained from 4 to 12 experiments.

### Assessment of DNL from [2-^14^C]acetic acid

DNL was assessed by measuring newly synthesized radiolabeled lipids from [2-^14^C]acetic acid, using a protocol adapted from Byrne et al. (2014). Briefly, culture medium was removed and cells were washed with warm PBS. Next, cells were incubated for 3 h with phenol red-free William’s E medium containing 1% fatty acid-free BSA, [2-^14^C]acetic acid (1850 Bq per well, corresponding to 19.4 pM) and 50 μM cold acetic acid. Medium was then gently removed and cells were washed with warm PBS before adding a mix of hexane/isopropanol (3:2; *v*/*v*). Cell culture plates were sealed and incubated for 1 h at room temperature for lipid extraction. After transfer of the plate content in 200-μL microtubes, hexane and PBS were added to have a hexane/isopropanol/PBS ratio of 6:2:3 (*v*/*v*/*v*). Microtubes were then centrifuged at 1000×*g* for 5 min and radiolabeled lipids were counted in the upper phase with a Tri-Carb 4910TR liquid scintillation counter (PerkinElmer). Results were normalized to total protein content and expressed in comparison to control cells.

### Measurement of apoB and apoC3 levels

Twenty-four hours after the last treatment, cell supernatants were removed and stored at − 80 °C until use for apolipoprotein measurement. ApoB level was measured using the Human Apolipoprotein B ELISA^pro^ kit from Mabtech (Nacka Strand, Sweden), according to the manufacturer’s instructions. Briefly, 50 μL of samples were diluted 2× in Apo ELISA buffer before a 2-h incubation in the precoated strip plates provided in the kit. The plates were washed and incubated 1 h with the biotinylated monoclonal anti-human apoB (LDL-11-biotin) antibody. The plates were then washed and incubated 1 h with a streptavidin–horseradish peroxidase conjugated antibody. After another washing, 3,3′,5,5′-tetramethylbenzidine (TMB) was used as substrate for 15 min before adding a stop solution. Absorbance at 450 nm was then measured using a POLARstar Omega microplate reader (BMG Labtech, Ortenberg). ApoB level was calculated utilizing a four-parameter standard curve, and normalized to the total cellular protein content and expressed in comparison to control cells.

Apolipoprotein C3 (apoC3) level was measured using the Human Apolipoprotein C3 kit from Cisbio Bioassays (Codolet, France), according to the manufacturer’s instructions. Briefly, 10 μL of supernatants was incubated with two different specific anti-human apoC3 antibodies, one donor and one acceptor. The detection principle of apoC3 is based on HTRF^®^ (Homogeneous Time Resolved Fluorescence), a time-resolved FRET (Förster Resonance Energy Transfer) technology. After 2 h of incubation, light emission was measured with the Infinite F Nano+ microplate reader (Tecan, Männedorf, Switzerland). ApoC3 level was calculated utilizing standard curve, based on the ratio of *A*_665 nm_/*A*_615 nm_, and normalized to the total cellular protein content. Results were expressed in comparison to control cells.

### Isolation of RNA and measurement of gene expression

Total RNA was extracted with the Nucleospin RNA isolation system purchased from Macherey-Nagel (Düren, Germany), which included a DNase treatment step. Quantification of isolated RNAs was assessed using a Nanodrop 1000 (Thermo Fisher Scientific, Waltham, MA). RNAs were reverse-transcribed into cDNAs using the High-Capacity cDNA Reverse Transcription Kit purchased from Applied Biosystems (Woolston, UK). Gene expression was assessed by real-time quantitative PCR analysis (RT-qPCR) using SYBR Green PCR Master Mix (Applied Biosystems) and a 384-well QuantStudio™ 7 Flex Real-Time PCR System (Thermo Fisher Scientific). Sequences of the primers used to measure gene expression are presented in Supplementary Table 3. Expression of *GAPDH* was chosen as reference and the 2^−ΔΔCt^ calculation method was used to express the relative expression of each selected gene.

### Protein extraction and Western blot analysis

Cells were lysed in ice-cold CelLytic M buffer (C2978; Sigma Aldrich) containing phosphatase and protease inhibitors from Roche Applied Science (Penzberg, Germany). Protein concentration was determined using the Pierce BCA Protein Assay Kit (Thermo Fisher Scientific) and equal amounts of protein were diluted with NuPAGE LDS Sample Buffer (Thermo Fisher Scientific). Samples (ca. 25 μg of proteins) were then separated by SDS-PAGE using NuPAGE 4–12% Bis-Tris gels (Thermo Fisher Scientific). Proteins were transferred to PVDF membranes (BioRad) and Ponceau S staining was used to confirm equal loading and transfer. Membranes were blocked in 7.5% milk in TBST (10 mM Tris–HCl, 100 mM NaCl, 0.02% Tween 20) for 1 h at room temperature and incubated overnight at 4 °C with antibodies against phospho-IRE1α (Ser724) (Abcam, ab124945), IRE1α (Cell Signaling, 3294), or HSC70 (Santa Cruz, sc-7298), whose dilutions were 1:1000. Membranes were washed with TBST, incubated for 1 h at room temperature with appropriate HRP secondary antibodies (Dako P0447 and P0448, respective dilutions 1:5000 and 1:10,000), washed with TBST, and then visualized by enhanced chemiluminescence (Pierce ECL Western Blotting Substrate; Thermo Fisher Scientific) using Fusion FX imaging system (Vilber Lourmat, Marne la Vallée, France). Protein content was quantified by densitometry with ImageJ software (National Institutes of Health, Bethesda, MD).

### Statistical analysis

All results are expressed as mean with standard deviation (SD). Statistical analyses were carried out using GraphPad Prism software V6.07. For each data analysis, the Kolmogorov–Smirnov normality test was performed. Comparisons between multiple groups were performed using one-way analysis of variance (ANOVA), followed by a Newman–Keuls multiple comparisons test, only when the normality test was positive. Comparisons between two groups were done using Student’s *t* test or Mann–Whitney *U* test when data were normally distributed or not, respectively.

## Results

### Human steatogenic drugs induce accumulation of neutral lipids in HepaRG cells and PHH

**Fig. 1:**
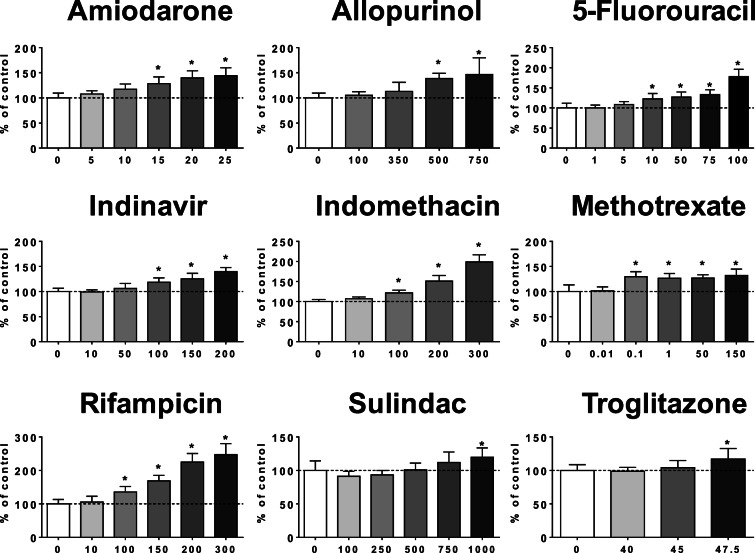
Effects of drugs on neutral lipids. HepaRG cells were treated for 4 consecutive days with different concentrations (μM) of amiodarone, allopurinol, 5-fluoruracil, indinavir, indomethacin, methotrexate, rifampicin, sulindac, and troglitazone. Results are means ± SD for 5 to 9 independent cultures. The horizontal dotted line represents 100% of the control values. Statistical significance of treated vs. control cells, determined by one-way ANOVA, is indicated by an asterisk (*P* < 0.05)

When HepaRG cells were treated for 4 days with the 12 selected steatogenic drugs, 9 of them induced a significant accumulation of neutral lipids, namely AMIO, ALLO, 5FU, INDI, INDO, METHO, SUL, RIF, and TRO (Fig. 1). Among these drugs, AMIO, ALLO, 5FU, INDI, INDO, RIF, and TRO also induced steatosis in at least one out of six PHH batches tested in this study (Supplementary Figs. 3 and 4). Interestingly, AMIO, used as positive control of steatogenic drug (Fromenty 2019; Fromenty and Pessayre 1995), was able to induce steatosis in the six different PHH batches (Supplementary Fig. 3). ALLO induced accumulation of neutral lipids in five PHH batches (Supplementary Fig. 3), whereas INDO was steatotic in two PHH batches (Supplementary Fig. 4). Lastly, 5FU, INDI, RIF, and TRO induced steatosis in only one PHH batch (Supplementary Fig. 4). In contrast, DPEN (100 to 2500 μM), METHI (250 to 1500 μM), and NIF (1 to 75 μM) did not induce steatosis in HepaRG cells (Supplementary Fig. 5), nor in the six different PHH batches (data not shown). However, DPEN and NIF induced in HepaRG cells a significant accumulation of neutral lipids for concentrations above 100 × *C*_max_ (Supplementary Fig. 5).

### Mechanisms of drug-induced steatosis in HepaRG cells

For drugs inducing neutral lipid accumulation in HepaRG cells, we next determined their respective effects on mtFAO, DNL, and VLDL secretion, as assessed by the measurement of apoB and apoC3 levels in the culture medium. In order to validate the methods set up in this study, we used different compounds that were previously shown to impair each metabolic pathway (Supplementary Fig. 6). To this end, mtFAO was blocked by etomoxir, a specific inhibitor of carnitine palmitoyltransferase-1 (CPT1) (Schreurs et al. 2010), which is the first enzyme mandatory for the import of long-chain fatty acids into mitochondria (Fromenty and Pessayre 1995). DNL was inhibited by a mixture of stearic and oleic acids (150 μM each), as already reported with other types of lipid overload (Ren et al. 2012; Wilson et al. 1990). Finally, apoB secretion was inhibited by lomitapide, a selective inhibitor of microsomal triglyceride transfer protein (MTP, encoded by the *MTTP* gene) (Hooper et al. 2015), which is an essential enzyme for the assembly and secretion of VLDL (Fisher et al. 2014; Yao and Wang 2012).

**Fig. 2:**
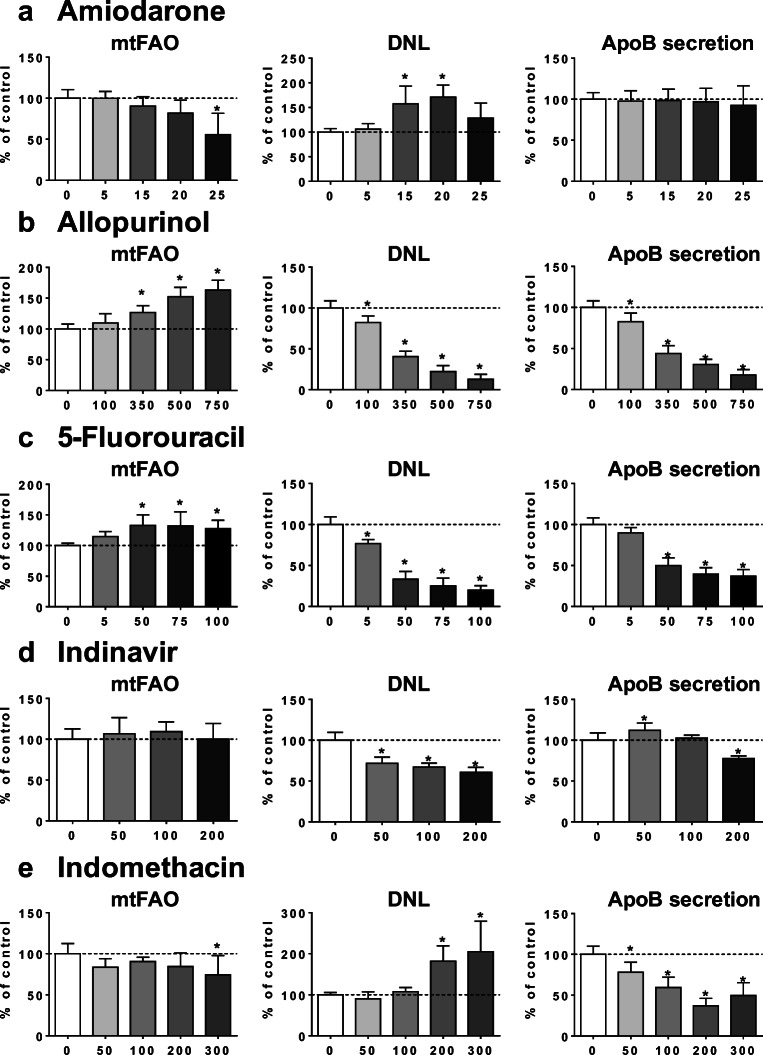
Effects of drugs on mtFAO, DNL, and apoB secretion. HepaRG cells were treated for 4 consecutive days with different concentrations (μM) of amiodarone (**a**), allopurinol (**b**), 5-fluoruracil (**c**), indinavir (**d**), or indomethacin (**e**) in order to determine their respective effects on mitochondrial fatty acid oxidation (mtFAO), de novo lipogenesis (DNL), and apoB secretion in the culture medium. Results are means ± SD for 5 to 9 independent cultures for mtFAO and DNL and 5 independent cultures for apoB secretion. The horizontal dotted line represents 100% of the control values. Statistical significance of treated vs. control cells, determined by one-way ANOVA, is indicated by an asterisk (*P* < 0.05)

**Fig. 3:**
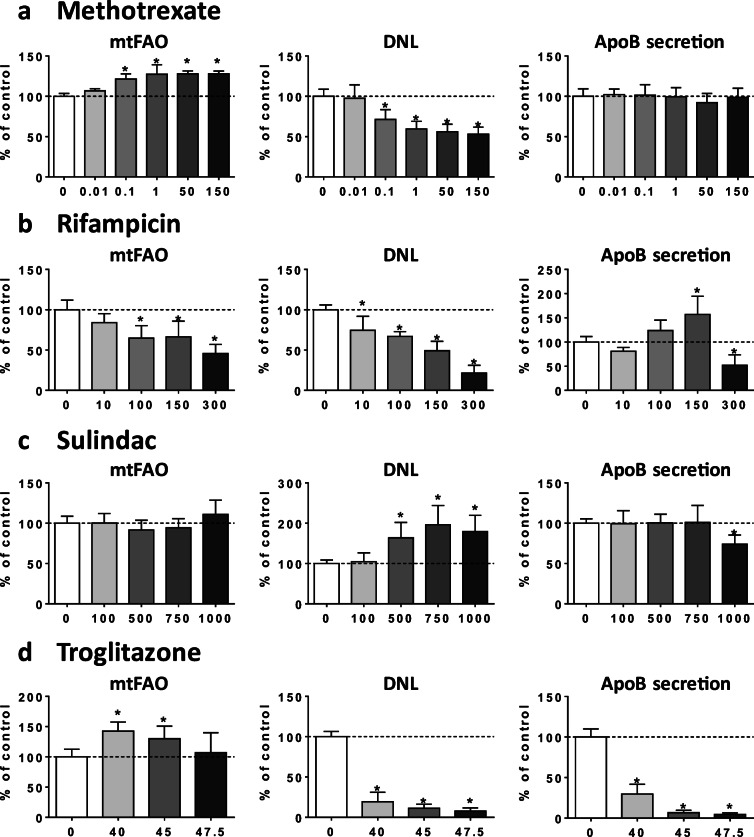
Effects of drugs on mtFAO, DNL, and apoB secretion. HepaRG cells were treated for 4 consecutive days with different concentrations (μM) of methotrexate (**a**), rifampicin (**b**), sulindac (**c**), or troglitazone (**d**) in order to determine their respective effects on mitochondrial fatty acid oxidation (mtFAO), de novo lipogenesis (DNL), and apoB secretion in the culture medium. Results are means ± SD for 5 to 9 independent cultures for mtFAO and DNL and 5 independent cultures for apoB secretion. The horizontal dotted line represents 100% of the control values. Statistical significance of treated vs. control cells, determined by one-way ANOVA, is indicated by an asterisk (*P* < 0.05)

In addition to AMIO, a prototypical inhibitor of mtFAO (Fromenty 2019; Fromenty and Pessayre 1995), INDO and RIF inhibited this metabolic pathway (Figs. 2 and 3). In contrast, ALLO, 5FU, METHO, and TRO were found to significantly enhance mtFAO (Figs. 2 and 3), possibly representing an adaptive response to steatosis. Three drugs (i.e., AMIO, INDO, and SUL) increased DNL, whereas ALLO, 5FU, INDI, METHO, RIF, and TRO were found to curb this metabolic pathway (Figs. 2 and 3). The latter effect might also represent an adaptation to lipid accumulation. Finally, ALLO, 5FU, INDI, INDO, RIF, SUL, and TRO reduced the secretion of both apoB (Figs. 2 and 3) and apoC3 (Supplementary Fig. 7), two important VLDL apolipoproteins. Noteworthy, secretion of apoB and apoC3 was dramatically decreased by 45 and 47.5 μM TRO treatment, resulting in less than 10% of the respective control (Fig. 3 and Supplementary Fig. 7).

### Mechanism of drug-induced inhibition of mtFAO

In this study, AMIO, INDO, and RIF reduced mtFAO in HepaRG cells as assessed with [U-^14^C]palmitic acid (Figs. 2 and 3). However, this method allows studying the whole mtFAO pathway and cannot identify the precise mechanism(s) whereby drugs can alter this metabolic process. Hence, additional investigations were carried out in permeabilized HepaRG cells incubated with different fatty acid derivatives. Experiments were also done with glutamate/malate because inhibition of MRC can secondarily impair mtFAO (Fromenty and Pessayre 1995; Fromenty et al. 1990b). EC_20_ data indicated that AMIO more specifically inhibited β-oxidation of the medium-chain fatty acid octanoyl-l-carnitine compared to other substrates (Table 1 and Supplementary Fig. 8). These data also indicated that AMIO-induced inhibition of long-chain fatty acids was more pronounced with palmitoyl-CoA + l-carnitine as compared to palmitoyl-carnitine (Table 1 and Supplementary Fig. 8). INDO more specifically inhibited oxygen consumption with palmitoyl-CoA + l-carnitine compared to the other substrates (Table 1 and Supplementary Fig. 8). Regarding RIF, all EC_20_ data were above 200 μM (Table 1), so that further investigations were performed in isolated mouse liver mitochondria incubated with this drug. In this model, EC_20_ were 20 ± 4, 16 ± 3, 17 ± 2, and 34 ± 12 μM, respectively, with palmitoyl-coenzyme A + l-carnitine, palmitoyl-l-carnitine, octanoyl-l-carnitine, and glutamate/malate (Supplementary Fig. 8).

**Table 1:** Ability of amiodarone, indomethacin and rifampicin to inhibit oxygen (O_2_) consumption in permeabilized HepaRG cells in the presence of different substrates

Drugs	EC_20_ values (μM) for inhibition of O_2_ consumption with different substrates			
	Palmitoyl-CoA + l-carnitine	Palmitoyl-l-carnitine	Octanoyl-l-carnitine	Glutamate + malate
Amiodarone (AMIO)	58 ± 10	79 ± 19	23 ± 7	93 ± 15
Indomethacin (INDO)	79 ± 8	163 ± 26	150 ± 11	119 ± 20
Rifampicin (RIF)	> 200	> 200	> 200	> 200

Effective concentration at 20% of the maximal effect (EC_20_) was determined as described in the “Materials and methods” section. Results are means ± SD for 2–3 independent experimental runs

### Mechanism of drug-induced activation of DNL

**Fig. 4:**
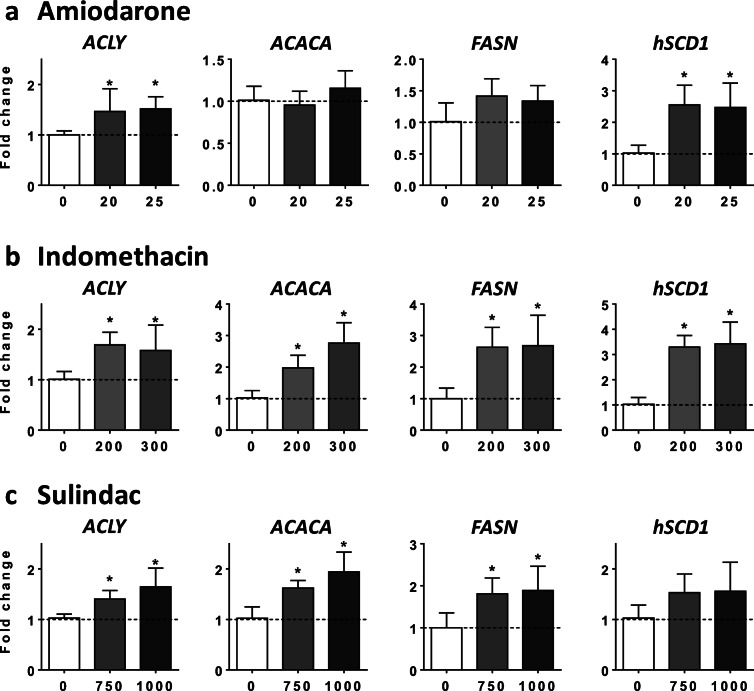
Effects of drugs on mRNA level of DNL enzymes. HepaRG cells were treated for 4 consecutive days with two concentrations (μM) of amiodarone (**a**), indomethacin (**b**), or sulindac (**c**) in order to determine their respective effects on the mRNA level of four enzymes playing a key role in DNL, namely ATP citrate lyase (*ACLY*), acetyl-CoA carboxylase alpha (*ACACA*), fatty acid synthase (*FASN*), and stearoyl-CoA desaturase 1 (*hSCD1*). Results are means ± SD for 5 independent cultures and are shown as fold change of control cells. The horizontal dotted line represents control values set at 1. Statistical significance of treated vs. control cells, determined by one-way ANOVA, is indicated by an asterisk (*P* < 0.05)

In this study, AMIO, INDO, and SUL stimulated DNL in HepaRG cells (Figs. 2 and 3). Thus, mRNA abundance of several key lipogenic genes was assessed in HepaRG cells treated with these drugs. AMIO enhanced mRNA level of ATP citrate lyase (*ACLY*) and stearoyl-CoA desaturase 1 (*hSCD1*) and tended to increase mRNA level of fatty acid synthase (*FASN*) (Fig. 4). Both INDO and SUL augmented mRNA abundance of *FASN*, *ACLY*, and acetyl-CoA carboxylase alpha (*ACACA*), whereas INDO also increased *hSCD1* mRNA level (Fig. 4).

### Mechanism of drug-induced impairment of VLDL secretion

**Fig. 5:**
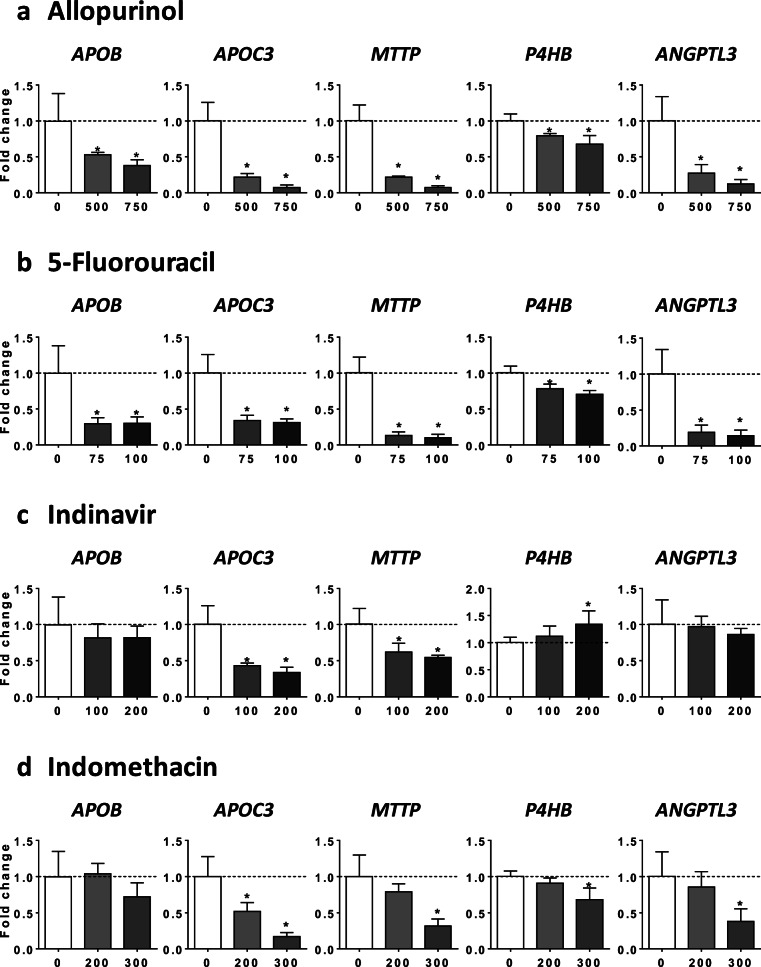
Effects of drugs on mRNA level of proteins and enzymes involved in VLDL assembly. HepaRG cells were treated for 4 consecutive days with two concentrations (μM) of allopurinol (**a**), 5-fluorouracil (**b**), indinavir (**c**), or indomethacin (**d**) in order to determine their respective effects on the mRNA level of five structural proteins and enzymes playing a significant role in VLDL assembly, namely, apolipoprotein B (*APOB*), apolipoprotein C3 (*APOC3*), microsomal triglyceride transfer protein (*MTTP*), prolyl 4-hydroxylase subunit beta (*P4HB*, also known as *PDI*), and angiopoietin-like 3 (*ANGPTL3*). Results are means ± SD for 5 independent cultures and are shown as fold change of control cells. The horizontal dotted line represents control values set at 1. Statistical significance of treated vs. control cells, determined by one-way ANOVA, is indicated by an asterisk (*P* < 0.05)

**Fig. 6:**
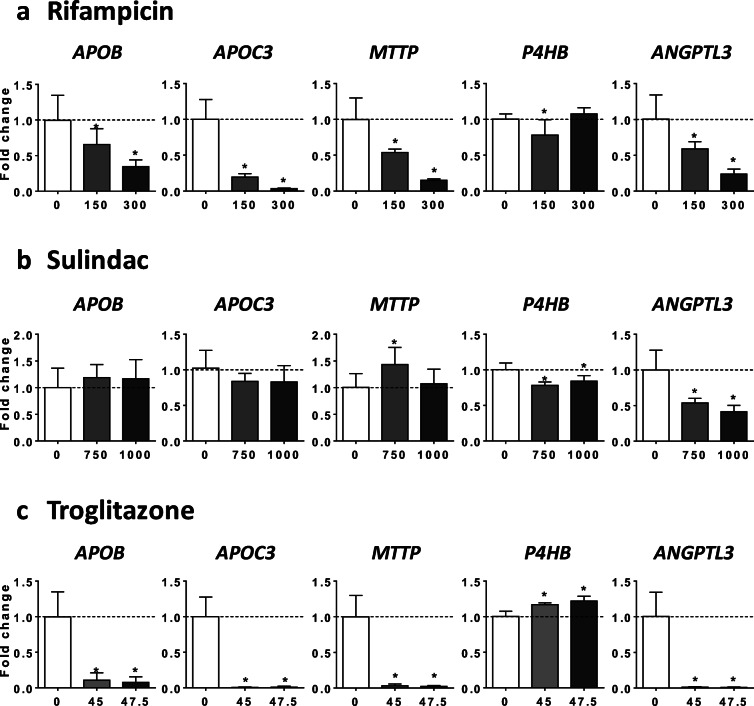
Effects of drugs on mRNA level of proteins and enzymes involved in VLDL assembly. HepaRG cells were treated for 4 consecutive days with two concentrations (μM) of rifampicin (**a**), sulindac (**b**), or troglitazone (**c**) in order to determine their respective effects on the mRNA level of five structural proteins and enzymes playing a significant role in VLDL assembly, namely, apolipoprotein B (*APOB*), apolipoprotein C3 (*APOC3*), microsomal triglyceride transfer protein (*MTTP*), prolyl 4-hydroxylase subunit beta (*P4HB*, also known as *PDI*), and angiopoietin-like 3 (*ANGPTL3*). Results are means ± SD for 5 independent cultures and are shown as fold change of control cells. The horizontal dotted line represents control values set at 1. Statistical significance of treated vs. control cells, determined by one-way ANOVA, is indicated by an asterisk (*P* < 0.05)

In this study, ALLO, 5FU, INDI, INDO, RIF, SUL, and TRO reduced VLDL secretion in HepaRG cells as assessed by measurement of apoB and apoC3 levels in the culture medium (Figs. 2 and 3 and Supplementary Fig. 7). In a first series of investigations, we determined the mRNA abundance of several structural proteins and enzymes playing a significant role in VLDL assembly, namely, apolipoprotein B (*APOB*), apolipoprotein C3 (*APOC3*), microsomal triglyceride transfer protein (*MTTP*), prolyl 4-hydroxylase subunit beta (*P4HB*, also known as protein disulfide isomerase family A member 1, *PDIA1* or *PDI*), and angiopoietin-like 3 (*ANGPTL3*) (Fisher et al. 2014; Wang et al. 2015; Yao and Wang 2012). Interestingly, the seven drugs decreased mRNA level of at least two of these genes regulating VLDL homeostasis (Figs. 5 and 6). In keeping with its strong effects on apoB and apoC3 secretion (Fig. 3 and Supplementary Fig. 7), TRO was found to nearly abolish the mRNA abundance of *APOB*, *APOC3*, *MTTP*, and *ANGPTL3* (Fig. 6).

**Fig. 7:**
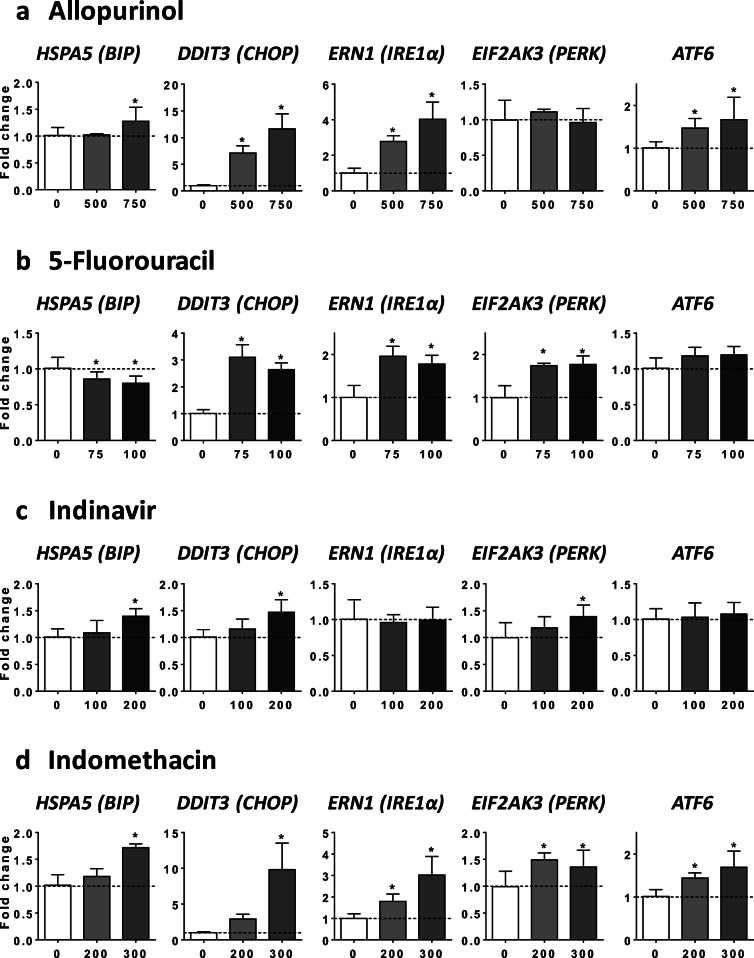
Effects of drugs on mRNA level of ER stress markers. HepaRG cells were treated for 4 consecutive days with two concentrations (μM) of allopurinol (**a**), 5-fluorouracil (**b**), indinavir (**c**), or indomethacin (**d**) in order to determine their respective effects on the mRNA level of five different proteins and transcription factors classically induced upon ER stress, namely heat shock protein family A member 5 (*HSPA5*, also known as *BIP*), DNA damage inducible transcript 3 (*DDIT3*, also known as *CHOP*), endoplasmic reticulum to nucleus signaling 1 (*ERN1*, also known as *IRE1α*), eukaryotic translation initiation factor 2 alpha kinase 3 (*EIF2AK3*, also known as *PERK*), and activating transcription factor 6 (*ATF6*). Results are means ± SD for 5 independent cultures and are shown as fold change of control cells. The horizontal dotted line represents control values set at 1. Statistical significance of treated vs. control cells, determined by one-way ANOVA, is indicated by an asterisk (*P* < 0.05)

**Fig. 8:**
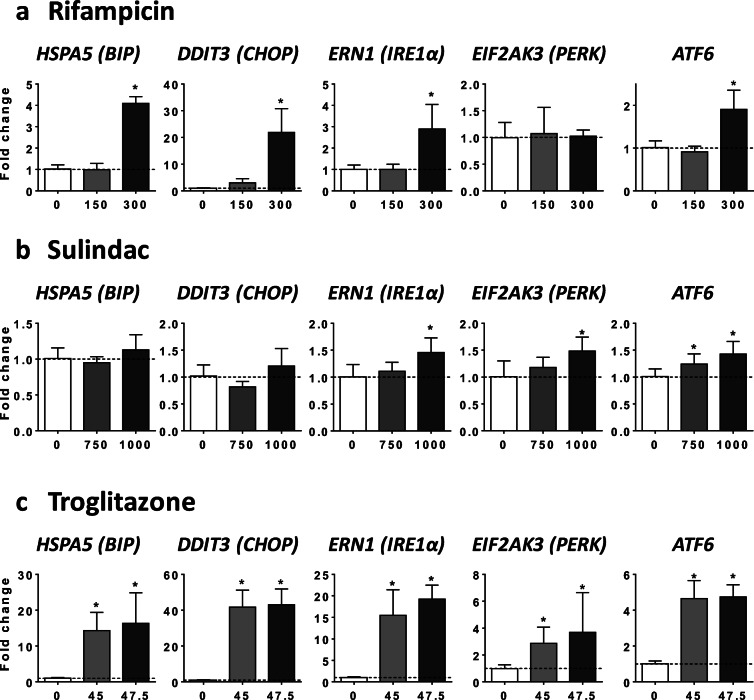
Effects of drugs on mRNA level of ER stress markers. HepaRG cells were treated for 4 consecutive days with two concentrations (μM) of rifampicin (**a**), sulindac (**b**), or troglitazone (**c**) in order to determine their respective effects on the mRNA level of five different proteins and transcription factors classically induced upon ER stress, namely heat shock protein family A member 5 (*HSPA5*, also known as *BIP*), DNA damage inducible transcript 3 (*DDIT3*, also known as *CHOP*), endoplasmic reticulum to nucleus signaling 1 (*ERN1*, also known as *IRE1α*), eukaryotic translation initiation factor 2 alpha kinase 3 (*EIF2AK3*, also known as *PERK*), and activating transcription factor 6 (*ATF6*). Results are means ± SD for 5 independent cultures and are shown as fold change of control cells. The horizontal dotted line represents control values set at 1. Statistical significance of treated vs. control cells, determined by one-way ANOVA, is indicated by an asterisk (*P* < 0.05)

Because previous investigations reported that endoplasmic reticulum (ER) stress-induced impairment of VLDL assembly and secretion could be associated with reduced mRNA level of *APOB*, *APOC3*, *MTTP*, and *ANGPTL3* (Feng et al. 2017; Rutkowski et al. 2008; Wang and Kaufman 2014; Yamamoto et al. 2010), we next determined whether the seven drugs were able to increase the expression of different transcription factors and proteins that are classically induced upon ER stress. To this end, we assessed the mRNA level of heat shock protein family A member 5 (*HSPA5*, also known as *BIP*), DNA damage inducible transcript 3 (*DDIT3*, also known as *CHOP*), endoplasmic reticulum to nucleus signaling 1 (*ERN1*, also known as *IRE1α*), eukaryotic translation initiation factor 2 alpha kinase 3 (*EIF2AK3*, also known as *PERK*), and activating transcription factor 6 (*ATF6*). Notably, the seven drugs enhanced the mRNA abundance of at least three of the five aforementioned genes, with *CHOP* and *IRE1α* being the most frequently increased (Figs. 7 and 8). We also tested the ability of INDO and RIF to activate IRE1α at the protein level. Consistent with the effect on *IRE1α* mRNA level (Figs. 7 and 8), both drugs increased protein level of phospho-IRE1α (pIRE1α) and total IRE1α (Supplementary Fig. 9).

**Fig. 9:**
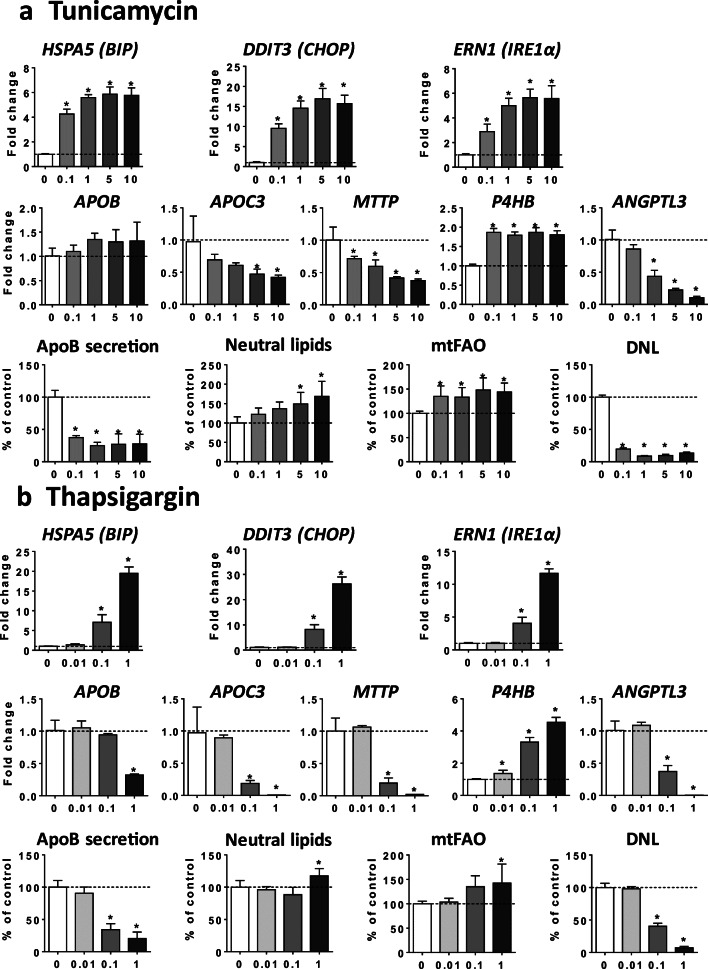
Effects of tunicamycin and thapsigargin on gene expression, apoB secretion, neutral lipids, mtFAO, and DNL. HepaRG cells were treated for 4 consecutive days with different concentrations (μM) of tunicamycin (**a**) or thapsigargin (**b**), two prototypical ER stress inducers, in order to determine their respective effects on the mRNA level of three proteins classically induced upon ER stress, namely heat shock protein family A member 5 (*HSPA5*, also known as *BIP*), DNA damage inducible transcript 3 (*DDIT3*, also known as *CHOP*), and endoplasmic reticulum to nucleus signaling 1 (*ERN1*, also known as *IRE1α*), mRNA level of five structural proteins and enzymes playing a significant role in VLDL assembly, namely, apolipoprotein B (*APOB*), apolipoprotein C3 (*APOC3*), microsomal triglyceride transfer protein (*MTTP*), prolyl 4-hydroxylase subunit beta (*P4HB*, also known as *PDI*), and angiopoietin-like 3 (*ANGPTL3*), apoB secretion in the culture medium, accumulation of neutral lipids, mitochondrial fatty acid oxidation (mtFAO), and de novo lipogenesis (DNL). Results are means ± SD for 3 independent cultures for mRNA abundance, 5 independent cultures for apoB secretion and neutral lipids and 5 to 6 independent cultures mtFAO and DNL. Results for mRNA level are shown as fold change of control cells. The horizontal dotted line represents control values set at 1 for gene expression, or 100% of the control values for other data. Statistical significance of treated vs. control cells, determined by one-way ANOVA, is indicated by an asterisk (*P* < 0.05)

In another series of experiments, HepaRG cells were treated with tunicamycin or thapsigargin, two prototypical inducers of ER stress (Foufelle and Fromenty 2016). As expected, both compounds augmented the mRNA level of *HSPA5* (*BIP*), *DDIT3* (*CHOP*), and *ERN1* (*IRE1α*) (Fig. 9). In addition, tunicamycin and thapsigargin reduced the mRNA level of *APOC3*, *MTTP*, and *ANGPTL3*, whereas the latter compound also reduced *APOB* mRNA abundance (Fig. 9). In contrast, expression of *P4HB* (*PDI*) was induced by both compounds (Fig. 9). These effects were associated with reduced secretion of apoB and increased neutral lipids (Fig. 9). Interestingly, both tunicamycin and thapsigargin increased mtFAO and strongly reduced DNL (Fig. 9).

**Fig. 10:**
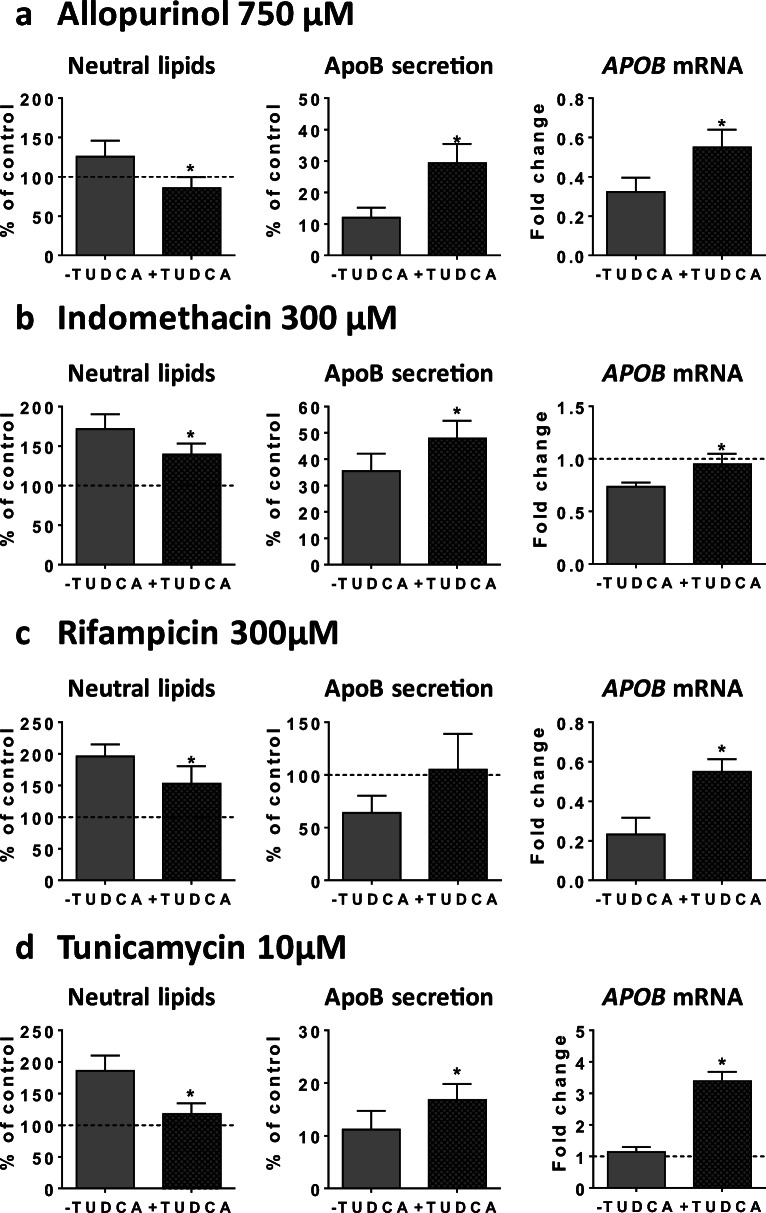
Effects of TUDCA on neutral lipids, apoB secretion, and *APOB* mRNA level in cells cotreated with allopurinol, indomethacin, rifampicin, and tunicamycin. HepaRG cells were treated for 4 consecutive days without (−) or with (+) TUDCA and with 750 μM allopurinol (**a**), 300 μM indomethacin (**b**), 300 μM rifampicin (**c**), or 10 μM tunicamycin (**d**). TUDCA effects were then determined for neutral lipids, apoB secretion, and *APOB* mRNA level. Results are means ± SD for 5 independent cultures for neutral lipids and 3 independent cultures for apoB secretion and *APOB* mRNA level. Results for mRNA level are shown as fold change of untreated control cells. The horizontal dotted line represents untreated control values set at 1 for gene expression, or 100% of the untreated control values for other data. Statistical significance of treated vs. control cells, determined by Student’s *t* test, is indicated by an asterisk (*P* < 0.05)

In a last series of experiments, investigations were carried out with TUDCA, a chemical chaperone known to alleviate ER stress in different pathophysiological conditions (Basseri and Austin 2012; Häussinger and Kordes, 2017). To this end, HepaRG cells were treated in absence or presence of TUDCA with 750 μM ALLO, 300 μM INDO, 300 μM RIF, or 10 μM tunicamycin. Our results indicated that TUDCA was able to alleviate neutral lipid accumulation induced by these four compounds (Fig. 10). This protective effect was associated with improved apoB secretion and higher apoB mRNA level. Of note, the effect of TUDCA alone has not been represented in Fig. 10 because, for an unknown reason, treatment of HepaRG cells with this compound led to an important increase in the number of nuclei (+ 38%), possibly reflecting cell proliferation. In contrast, such increase was not observed when TUDCA was associated with ALLO, INDO, RIF, or tunicamycin.

## Discussion

Many drugs can induce hepatic steatosis (Amacher and Chalasani 2014; Fromenty and Pessayre 1995; Satapathy et al. 2015; Fromenty 2019). Whereas profound mitochondrial dysfunction is involved in rare individuals with predisposing factors, most cases of steatosis are benign (Amacher and Chalasani 2014; Fromenty 2019; Fromenty and Pessayre 1995). Nevertheless, if the treatment is not discontinued, this lesion can progress in some patients to steatohepatitis and even to cirrhosis (Amacher and Chalasani 2014; Fromenty 2019; Massart et al. 2013; Satapathy et al. 2015). Hence, it is important to better understand the mechanism(s) whereby drugs can induce steatosis in the absence of severe mitochondrial dysfunction. Previous studies suggested the involvement of mild-to-moderate inhibition of mtFAO, increased DNL, and impairment of VLDL secretion (Fromenty 2019; Begriche et al. 2011; Lettéron et al. 2003; Tolosa et al. 2016; Grünig et al. 2018; Rooney et al. 2019).

In this study, we investigated 12 drugs able to induce steatosis in patients, namely AMIO (used as positive control), ALLO, DPEN, 5FU, INDI, INDO, METHI, METHO, NIF, RIF, SUL, and TRO (Fromenty 2019; Biour et al. 2004; Wang et al. 2013). Notably, working drug concentrations were chosen in order to induce only slight to moderate loss of cellular ATP (i.e., ATP level decreased by less than 30% as compared to control) and were usually beneath 100 × *C*_max_ (Supplementary Table 1). Nonetheless, these working concentrations were in general above the therapeutic concentrations measured in blood (or plasma) of treated patients (Supplementary Table 2). Thus, drug-induced steatosis and metabolic alterations observed in this study might preferentially occur in patients with overdose or drug–drug interactions, or in individuals with genetic predispositions or pre-existing liver diseases (Begriche et al. 2011; Fromenty and Pessayre 1995).

Among the 12 drugs, AMIO, ALLO, 5FU, INDI, INDO, METHO, SUL, RIF, and TRO induced steatosis in HepaRG cells, thus confirming that the HepaRG cell line represents a suitable model in order to study drug-induced steatosis (Anthérieu et al. 2011; Cuykx et al. 2018; Tolosa et al. 2016). In addition, we found that AMIO, ALLO, 5FU, INDI, INDO, RIF, and TRO also induced steatosis in at least one out of six different PHH batches. In contrast, DPEN, METHI, and NIF did not induce steatosis in HepaRG cells, nor in the six PHH batches. We did not perform further experiments in order to determine why these drugs were unable to induce steatosis in HepaRG cells and PHH for the selected concentrations. It is possible that higher concentrations and/or longer exposure would be necessary to induce triglyceride accumulation in cultured cells. In keeping with this assumption, 100 μM NIF and 5000 μM DPEN induced steatosis in HepaRG cells (Supplementary Fig. 5), thus above 100 × *C*_max_. Alternatively, these drugs might induce hepatic steatosis via an indirect mechanism such as insulin resistance and hyperinsulinemia (Begriche et al. 2011; Fromenty 2019).

Of note, we arbitrarily considered that a reduction of ATP level by less than 30% as compared to control reflected a lack of severe mitochondrial dysfunction. Nevertheless, although there is no consensual definition of what can be considered as severe mitochondrial dysfunction, previous investigations in primary cultured hepatocytes and rodent liver reported the occurrence of drug-induced severe (or profound) mitochondrial dysfunction whenever ATP levels felt below 50–60% of the control values (González et al. 2009; Knight and Jaeschke 2002; Lee et al. 2013). In some experimental conditions, we cannot exclude the possibility that mitochondrial dysfunction might have been underestimated as a consequence of glycolysis-driven ATP production. However, it should be underlined that when a direct assessment of mitochondrial function was performed by measuring mtFAO with [U-^14^C]palmitic acid, drug-induced inhibition of this metabolic pathway was either mild (INDO) or moderate (AMIO and RIF). Lastly, the slight or moderate decrease in the number of living cells, as assessed with the Hoechst 33342 dye (Supplementary Fig. 10), was also in favor of the absence of severe mitochondrial dysfunction.

For the nine drugs inducing steatosis in HepaRG cells, further experiments were carried out to determine the mechanism(s) of triglyceride accumulation in the absence of severe mitochondrial dysfunction. Several important conclusions could be drawn from these investigations: (1) inhibition of mtFAO and activation of DNL were not frequently observed; (2) reduced VLDL secretion was more frequently involved, possibly as a consequence of reduced mRNA abundance of different proteins and enzymes playing a significant role in VLDL assembly, in particular *APOB*, *APOC3*, *MTTP*, and *ANGPTL3* (Fisher et al. 2014; Wang et al. 2015; Yao and Wang 2012); (3) these effects were associated with ER stress as assessed by mRNA level of *BIP*, *CHOP*, *IRE1α*, *PERK*, and *ATF6*; (4) the prototypical inducers of ER stress tunicamycin and thapsigargin (Foufelle and Fromenty 2016) reduced mRNA abundance of *APOC3*, *MTTP*, and *ANGPTL3*, decreased apoB secretion, and induced accumulation of neutral lipids; (5) the chemical chaperone TUDCA was able to partially prevent steatosis and apoB secretion impairment induced by tunicamycin, ALLO, INDO, and RIF.

In addition to AMIO used as positive control of drug-induced inhibition of mtFAO (Fromenty et al. 1990a; Spaniol et al. 2001; Serviddio et al. 2011), only INDO and RIF impaired oxidation of [U-^14^C]palmitic acid in HepaRG cells. Further investigations in permeabilized HepaRG cells reveal that inhibition of oxygen consumption by AMIO is more pronounced with octanoyl-l-carnitine than with other mtFAO substrates. This confirms our previous investigations in mice showing that this antiarrhythmic drug strongly impaired the β-oxidation of medium-chain fatty acids (Fromenty et al. 1990b). In addition, our results suggest that AMIO could inhibit CPT1 since the impairment of oxygen consumption is more pronounced with palmitoyl-CoA + l-carnitine when compared to palmitoyl-carnitine. This hypothesis is in keeping with previous studies demonstrating that AMIO was a CPT1 inhibitor (Hamdan et al. 2001; Kennedy et al. 1996). Finally, our results confirm that low concentrations of AMIO directly inhibit the mtFAO pathway itself, whereas higher concentrations are required to impair MRC (Fromenty et al. 1990a, b). Interestingly, our results in permeabilized HepaRG cells also suggested that INDO could inhibit CPT1. Finally, the EC_20_ profile found with RIF in isolated mouse liver mitochondria suggested that this antibiotic could directly impair mtFAO process itself and not as a secondary consequence of MRC impairment. Moreover, RIF might induce mtFAO impairment through a mechanism independent of CPT1 and the length of fatty acids. Indeed, the respective EC_20_ found with the three fatty acid derivatives was about similar. To the best of our knowledge, our results regarding INDO- and RIF-induced inhibition of mtFAO in hepatic cells have not been reported thus far.

In this study, AMIO, INDO, and SUL stimulated DNL in HepaRG cells. AMIO enhanced mRNA level of *ACLY* and *hSCD1*, whereas both INDO and SUL augmented mRNA abundance of *FASN*, *ACLY*, and *ACACA*. In addition, INDO also increased *hSCD1* mRNA level. Thus, these results suggest that AMIO, INDO, and SUL activate one or several transcription factors positively regulating DNL. In keeping with this hypothesis, a previous study in our laboratory reported that, after 14 days, AMIO-induced steatosis in HepaRG cells was associated with an increased mRNA abundance of sterol regulatory element binding transcription factor 1 (*SREBF1*, also known as *SREBP1*) and other lipogenic genes including *FASN* and thyroid hormone responsive (*THRSP*, also known as *SPOT14*) (Anthérieu et al. 2011). Moreover, recent investigations performed in HeLa cell transactivation assays showed that INDO and sulindac sulfide could activate the lipogenic transcription factor peroxisome proliferator-activated receptor gamma (PPARγ) (Puhl et al. 2015). Finally, investigations performed in rats suggested that AMIO and SUL could activate hepatic SREBP in this animal species (Corton 2019).

In contrast, ALLO, 5FU, INDI, METHO, RIF, and TRO significantly reduced DNL in HepaRG cells. Interestingly, recent data suggested that ALLO and 5FU might decrease DNL (Sommer et al. 2017; García-Arroyo et al., 2019). However, previous investigations reported increased DNL with INDI (Lenhard et al. 2000), RIF (Huang et al. 2016), and TRO (Schadinger et al. 2005). The discrepancies between these investigations and ours might be due to differences in the respective experimental models and conditions including durations of treatment and methods to assess DNL.

Many xenobiotics including drugs can induce ER stress in liver (Chen et al. 2014; Dara et al. 2011; Foufelle and Fromenty 2016). However, except for a few compounds, the involved mechanisms are still unknown (Chen et al. 2014; Foufelle and Fromenty 2016). Notably, mechanisms of ER stress are difficult to investigate because many events could be involved such as impairment of protein glycosylation, inhibition of the sarcoplasmic reticulum Ca^2+^-ATPase (SERCA), covalent binding of reactive metabolites to ER proteins, proteasome inhibition, oxidative stress (with subsequent oxidative damage of key ER components), activation of mitogen-activated protein kinase (MAPK), severe mitochondrial dysfunction, and increased cytosolic calcium (Chen et al. 2014; Dara et al. 2011; Foufelle and Fromenty 2016). Nevertheless, despite this multitude of cues, previous studies consistently demonstrate that ER stress can induce hepatic steatosis (Baiceanu et al. 2016; Dara et al. 2011; Foufelle and Fromenty 2016; Wang and Kaufman 2014). Although the mechanisms whereby ER stress induces hepatic lipid accumulation actually seem to depend on the ER stressors, inhibition of VLDL (or apoB) secretion was frequently reported in different conditions of ER stress including after tunicamycin treatment (Feng et al. 2017; Foufelle and Fromenty 2016; Ota et al. 2008; Qiu et al. 2009; Rutkowski et al. 2008). In this study, both tunicamycin (which impairs protein glycosylation) and thapsigargin (which inhibits SERCA) reduced apoB secretion and induced accumulation of neutral lipids in HepaRG cells. The effect on VLDL secretion seems pivotal for lipid accumulation in our experimental conditions since both compounds strongly inhibited DNL and stimulated mtFAO. Finally, the role of ER stress in decreased apoB secretion and steatosis was further strengthened by our observation that TUDCA partially prevented these deleterious effects when cells were treated with tunicamycin, ALLO, INDO and RIF.

ER stress might impair VLDL assembly and secretion by different mechanisms. A first mechanism, in line with our results, could be lower mRNA level of key proteins involved in VLDL assembly including *APOB*, *APOC3*, *MTTP*, and *ANGPTL3* (Feng et al. 2017; Rutkowski et al. 2008; Wang and Kaufman 2014; Yamamoto et al. 2010). Notably, ANGPTL3 mRNA could be degraded via regulated IRE1α-dependent decay (RIDD) (Maurel et al. 2014; Wang and Kaufman 2014). Indeed, IRE1α presents an endoribonuclease activity able to degrade many endogenous mRNAs (Maurel et al. 2014; Oikawa et al. 2010). In our study, enhanced IRE1α mRNA level was observed with ALLO, 5FU, INDO, SUL, RIF, and TRO. Furthermore, at the protein level, both INDO and RIF increased pIRE1α and total IRE1α. However, it should be underlined that ER stress could reduce the level of mRNAs that are indirect RIDD substrates (Maurel et al. 2014) or via some unspecific mechanisms unrelated to the unfolded protein response (UPR) (Bergmann et al. 2018). Another nonexclusive mechanism could be ER stress-mediated apoB degradation through both proteasomal and non-proteasomal pathways (Ota et al. 2008; Qiu et al. 2009, 2011). Thus, further investigations are required in order to determine the exact mechanism(s) involved.

While our study suggests that impairment of VLDL secretion is frequently involved in drug-induced steatosis without severe mitochondrial dysfunction, we acknowledge that more compounds should be studied in order to confirm this hypothesis. Nevertheless, numerous drugs induce ER stress (Chen et al. 2014; Foufelle and Fromenty 2016), which in turn can impair VLDL assembly by various mechanisms (Fisher et al. 2014). For instance, ER stress triggers apoB degradation via proteasomal and nonproteasomal pathways (Qiu et al. 2009, 2011), or might induce a reduction of apoB mRNA levels as suggested by our results. Notably, drug-induced reduction of VLDL secretion can cause a limitation of clinical use because of high risk of hepatic steatosis, as previously reported with lomitapide and mipomersen, an antisense oligonucleotide directed against apoB-100 (Gouni-Berthold and Berthold, 2015; Hooper et al. 2015). Hence, if our data are extended to a larger number of drugs, it would be important in the future to include during preclinical safety studies a systematic exploration of the hepatic VLDL secretion pathway. This is easily feasible with high-throughput measurement of apoB and apoC3 levels in culture media. Moreover, for drugs that are already on the market, recommendations should be done regarding the association of pharmaceuticals impairing VLDL output. Warning should also be expressed with respect to prescription of such compounds in patients with pre-existing liver disease featuring fatty liver or steatohepatitis such as NAFLD, alcoholic liver disease, and chronic hepatitis C virus infection.

Among the nine drugs inducing steatosis in HepaRG cells, METHO was the only drug for which we could not find the mechanism(s) of lipid accumulation in HepaRG cells. Indeed, METHO did not reduce mtFAO and apoB secretion, nor it did enhance DNL. However, 50 and 150 μM METHO significantly increased by 60% and 55% the mRNA level of fatty acid translocase (FAT, also known as CD36) (Supplementary Fig. 11). FAT/CD36 mRNA level was also significantly augmented by AMIO and INDO (Supplementary Fig. 11), but future investigations will be required to determine whether these drugs also increase the activity of this translocase. Enhanced FAT/CD36 expression and activity plays a major role in higher hepatic fatty acid uptake and fatty liver in different pathophysiological situations (He et al. 2011; Zhang et al. 2018). Previous studies also suggested that steatosis induced by RIF, tetracycline, and valproic acid could be, at least in part, secondary to increased FAT/CD36 expression or activity (Benet et al. 2014; Choi et al. 2015; Huang et al. 2016), although this effect was not observed with RIF in our study (Supplementary Fig. 11). Nonetheless, these investigations and ours strengthen the concept that for exposure not inducing severe mitochondrial dysfunction drugs can induce steatosis through different mechanisms.

Finally, it is noteworthy that ALLO, 5FU, and TRO-induced impairment of apoB secretion was associated with increased mtFAO and reduced DNL in a similar manner than tunicamycin and thapsigargin. This suggests that common metabolic adaptations may occur to limit lipid accumulation induced by some ER stressors. Whether drugs which inhibit mtFAO (AMIO, INDO, RIF) and/or activate DNL (AMIO, INDO, SUL) impair these metabolic adaptations warrants further study.

## Electronic supplementary material

ESM 1 (PPTX 12687 kb)

ESM 2 (DOCX 27 kb)

## Abbreviations

ALLO: Allopurinol
AMIO: Amiodarone
ApoB: Apolipoprotein B
ApoC3: Apolipoprotein C3
CPT1: Carnitine palmitoyltransferase-1
DILI: Drug-induced liver injury
DNL: De novo Lipogenesis
DPEN: d-Penicillamine
ER: Endoplasmic reticulum
5FU: 5-Fluorouracil
INDI: Indinavir
INDO: Indomethacin
METHI: Methimazole
METHO: Methotrexate
MRC: Mitochondrial respiratory chain
mtFAO: Mitochondrial fatty acid oxidation
NIF: Nifedipine
PPAR: Peroxisome proliferator-activated receptor
RIF: Rifampicin
PHH: Primary human hepatocytes
SUL: Sulindac
TRO: Troglitazone
VLDL: Very low-density lipoprotein
