# Supplementary material for: Drug-induced hepatic steatosis in absence of severe mitochondrial dysfunction in HepaRG cells: proof of multiple mechanism-based toxicity
Source: Cell Biol Toxicol. 2020 Jun 14;37(2):151–75. doi: 10.1007/s10565-020-09537-1 (PMC8012331; doi:10.1007/s10565-020-09537-1)
Supplement: Supplementary file 1 — (PPTX 12687 kb) [file 10565_2020_9537_MOESM1_ESM.pptx]

## Slide 1
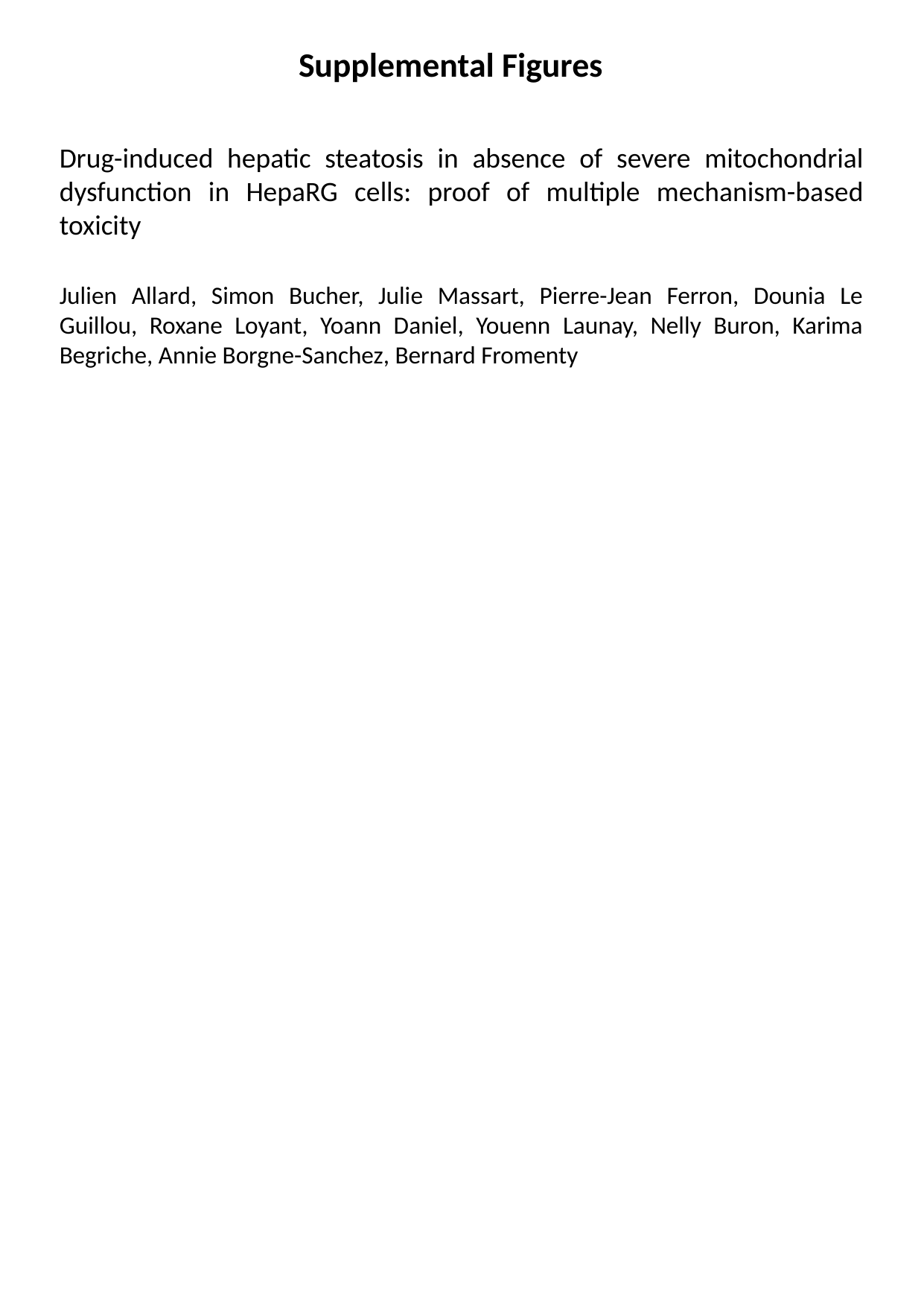

Supplemental Figures
Drug-induced hepatic steatosis in absence of severe mitochondrial dysfunction in HepaRG cells: proof of multiple mechanism-based toxicity
Julien Allard, Simon Bucher, Julie Massart, Pierre-Jean Ferron, Dounia Le Guillou, Roxane Loyant, Yoann Daniel, Youenn Launay, Nelly Buron, Karima Begriche, Annie Borgne-Sanchez, Bernard Fromenty

## Slide 2
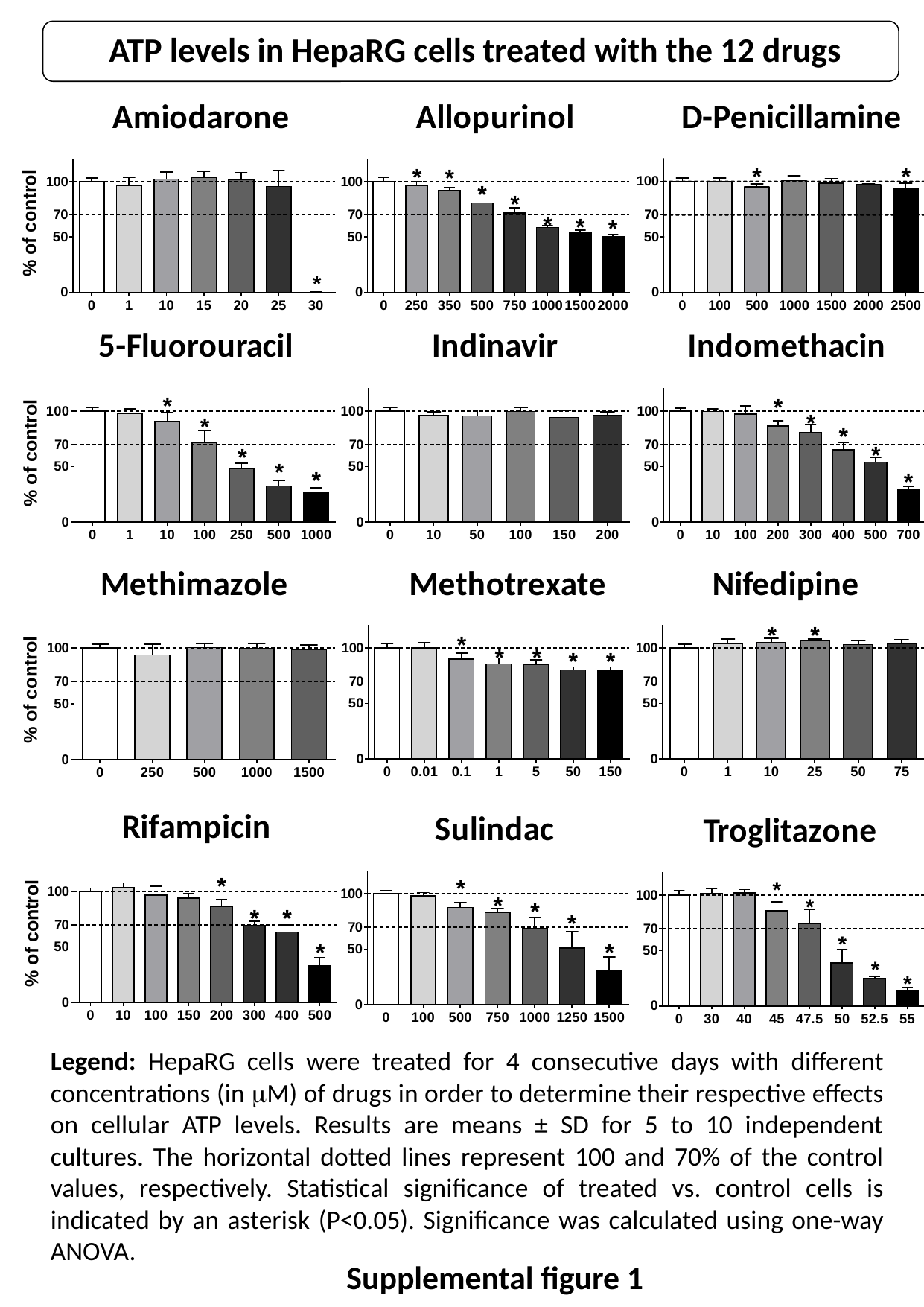

ATP levels in HepaRG cells treated with the 12 drugs
Legend: HepaRG cells were treated for 4 consecutive days with different concentrations (in mM) of drugs in order to determine their respective effects on cellular ATP levels. Results are means ± SD for 5 to 10 independent cultures. The horizontal dotted lines represent 100 and 70% of the control values, respectively. Statistical significance of treated vs. control cells is indicated by an asterisk (P<0.05). Significance was calculated using one-way ANOVA.
Supplemental figure 1

## Slide 3
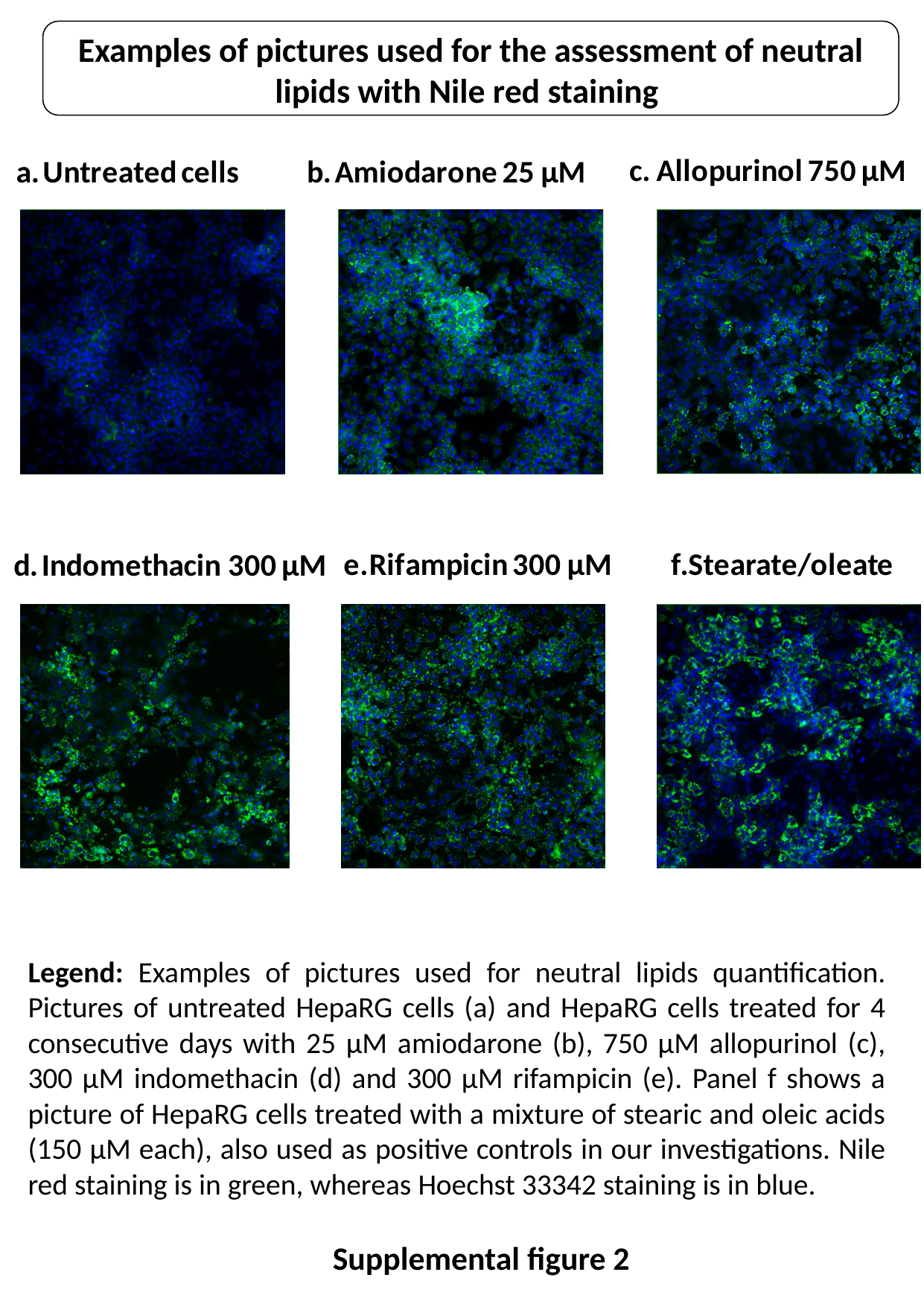

Examples of pictures used for the assessment of neutral lipids with Nile red staining
Legend: Examples of pictures used for neutral lipids quantification. Pictures of untreated HepaRG cells (a) and HepaRG cells treated for 4 consecutive days with 25 µM amiodarone (b), 750 µM allopurinol (c), 300 µM indomethacin (d) and 300 µM rifampicin (e). Panel f shows a picture of HepaRG cells treated with a mixture of stearic and oleic acids (150 µM each), also used as positive controls in our investigations. Nile red staining is in green, whereas Hoechst 33342 staining is in blue.
Supplemental figure 2

## Slide 4
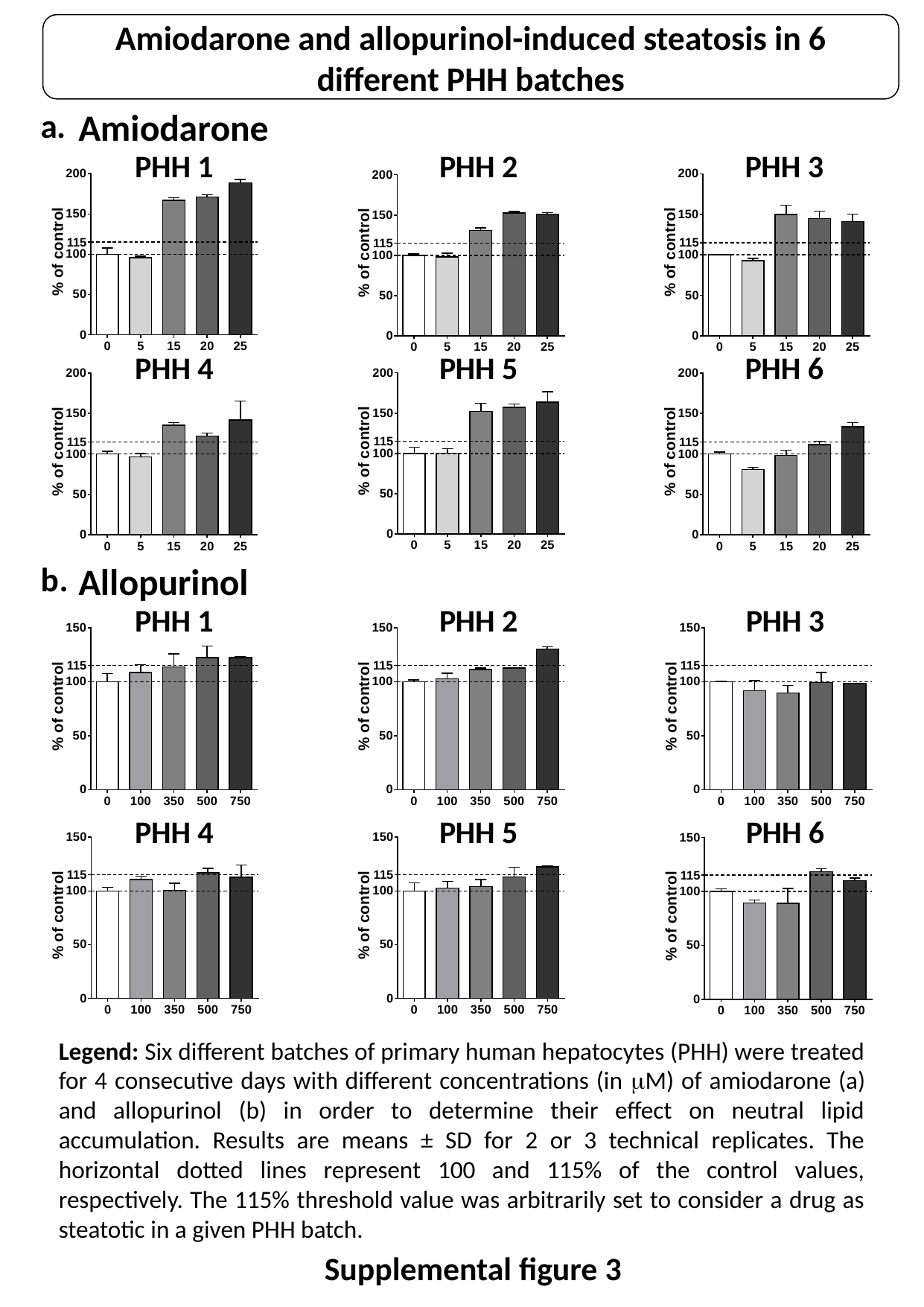

Amiodarone and allopurinol-induced steatosis in 6 different PHH batches
Legend: Six different batches of primary human hepatocytes (PHH) were treated for 4 consecutive days with different concentrations (in mM) of amiodarone (a) and allopurinol (b) in order to determine their effect on neutral lipid accumulation. Results are means ± SD for 2 or 3 technical replicates. The horizontal dotted lines represent 100 and 115% of the control values, respectively. The 115% threshold value was arbitrarily set to consider a drug as steatotic in a given PHH batch.
Supplemental figure 3

## Slide 5
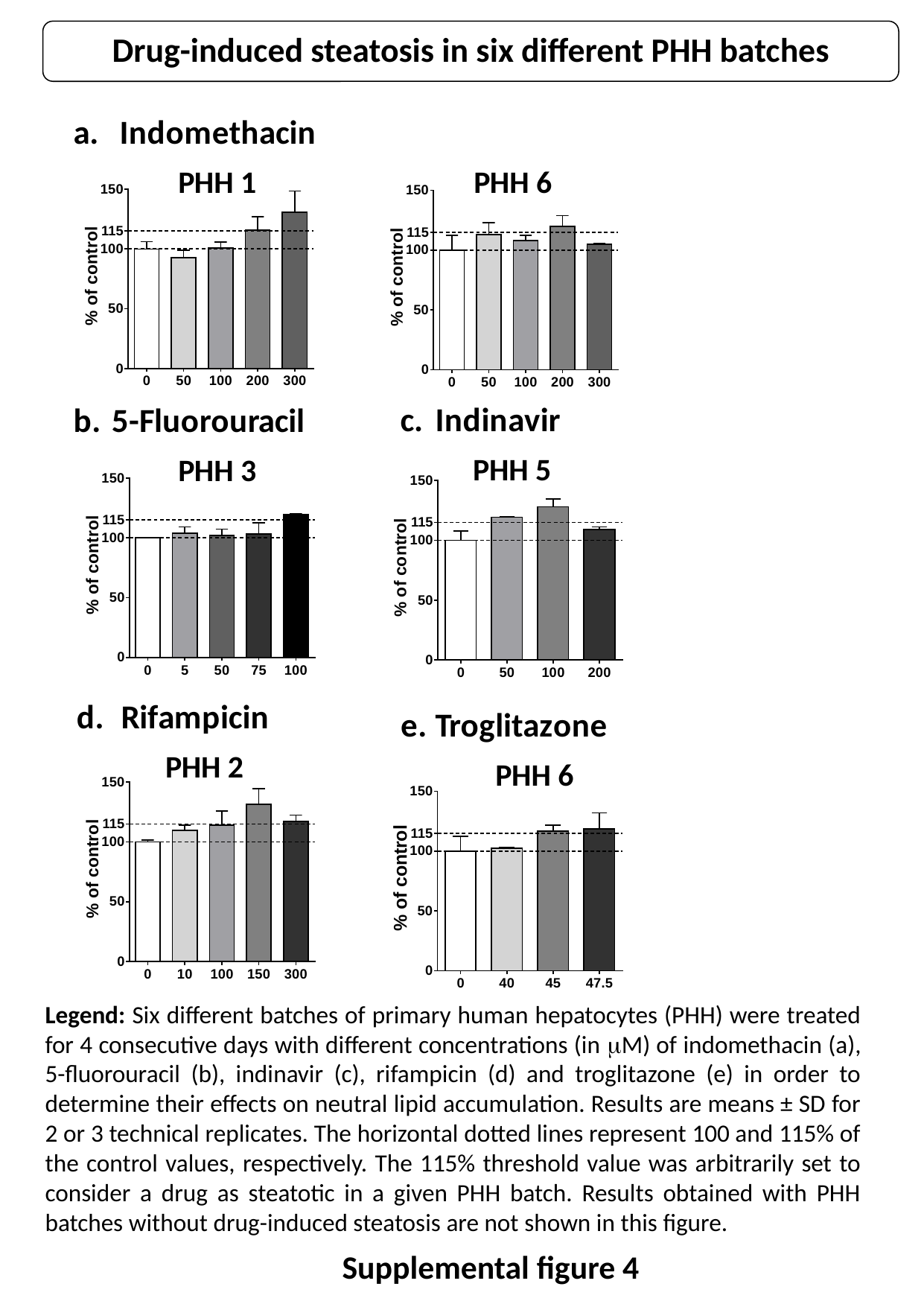

Drug-induced steatosis in six different PHH batches
Legend: Six different batches of primary human hepatocytes (PHH) were treated for 4 consecutive days with different concentrations (in mM) of indomethacin (a), 5-fluorouracil (b), indinavir (c), rifampicin (d) and troglitazone (e) in order to determine their effects on neutral lipid accumulation. Results are means ± SD for 2 or 3 technical replicates. The horizontal dotted lines represent 100 and 115% of the control values, respectively. The 115% threshold value was arbitrarily set to consider a drug as steatotic in a given PHH batch. Results obtained with PHH batches without drug-induced steatosis are not shown in this figure.
Supplemental figure 4

## Slide 6
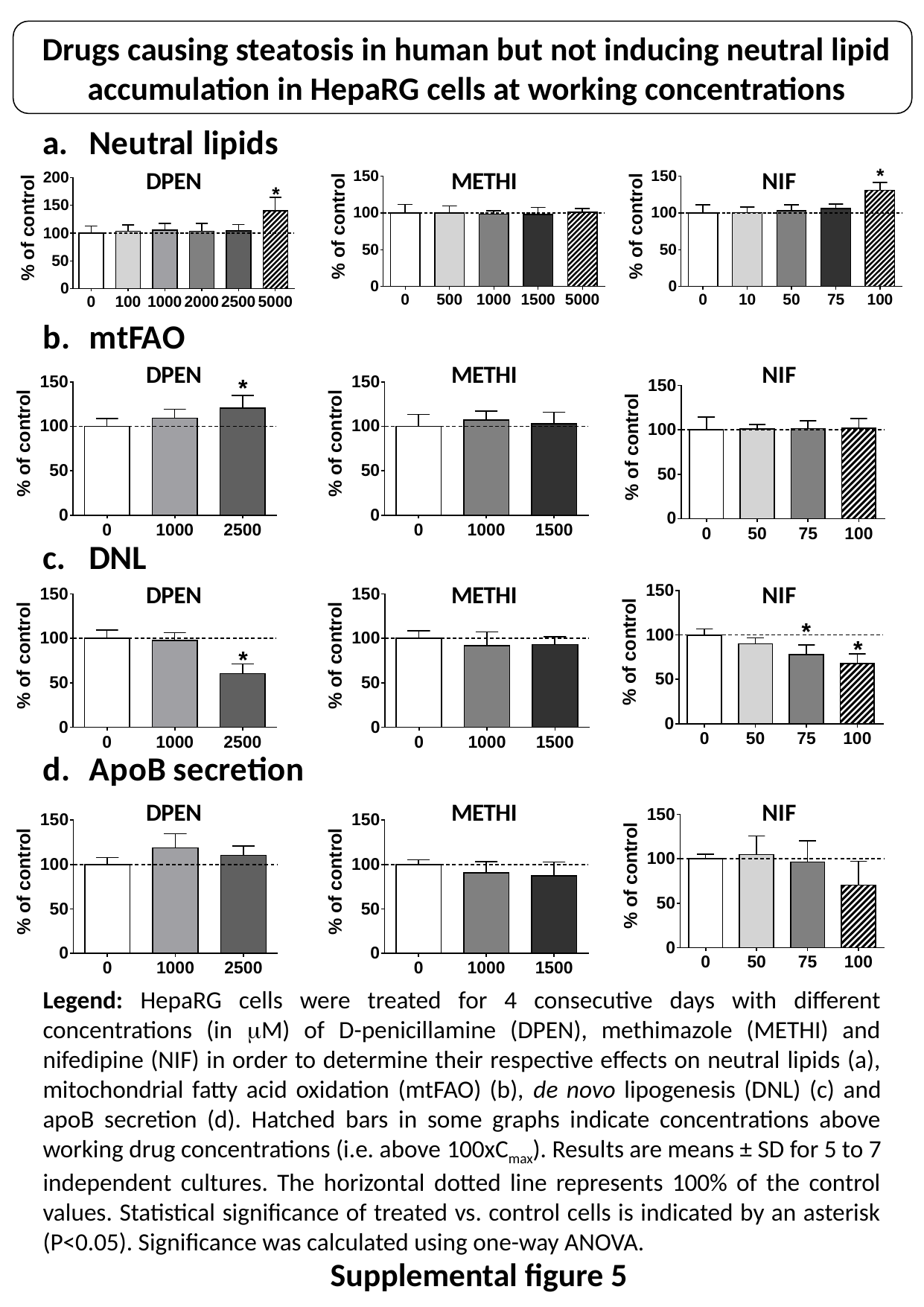

Drugs causing steatosis in human but not inducing neutral lipid accumulation in HepaRG cells at working concentrations
Legend: HepaRG cells were treated for 4 consecutive days with different concentrations (in mM) of D-penicillamine (DPEN), methimazole (METHI) and nifedipine (NIF) in order to determine their respective effects on neutral lipids (a), mitochondrial fatty acid oxidation (mtFAO) (b), de novo lipogenesis (DNL) (c) and apoB secretion (d). Hatched bars in some graphs indicate concentrations above working drug concentrations (i.e. above 100xCmax). Results are means ± SD for 5 to 7 independent cultures. The horizontal dotted line represents 100% of the control values. Statistical significance of treated vs. control cells is indicated by an asterisk (P<0.05). Significance was calculated using one-way ANOVA.
Supplemental figure 5

## Slide 7
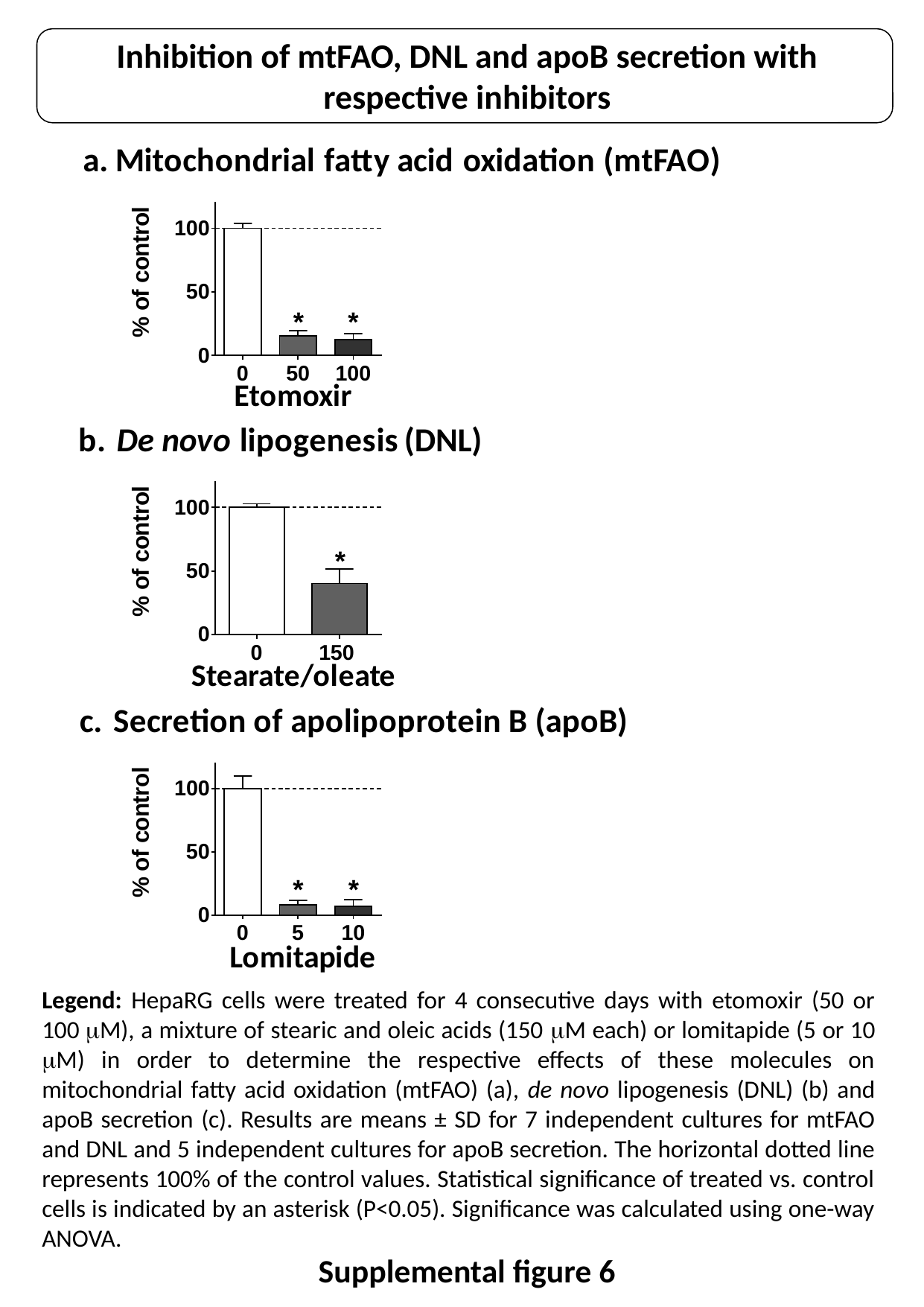

Inhibition of mtFAO, DNL and apoB secretion with respective inhibitors
Legend: HepaRG cells were treated for 4 consecutive days with etomoxir (50 or 100 mM), a mixture of stearic and oleic acids (150 mM each) or lomitapide (5 or 10 mM) in order to determine the respective effects of these molecules on mitochondrial fatty acid oxidation (mtFAO) (a), de novo lipogenesis (DNL) (b) and apoB secretion (c). Results are means ± SD for 7 independent cultures for mtFAO and DNL and 5 independent cultures for apoB secretion. The horizontal dotted line represents 100% of the control values. Statistical significance of treated vs. control cells is indicated by an asterisk (P<0.05). Significance was calculated using one-way ANOVA.
Supplemental figure 6

## Slide 8
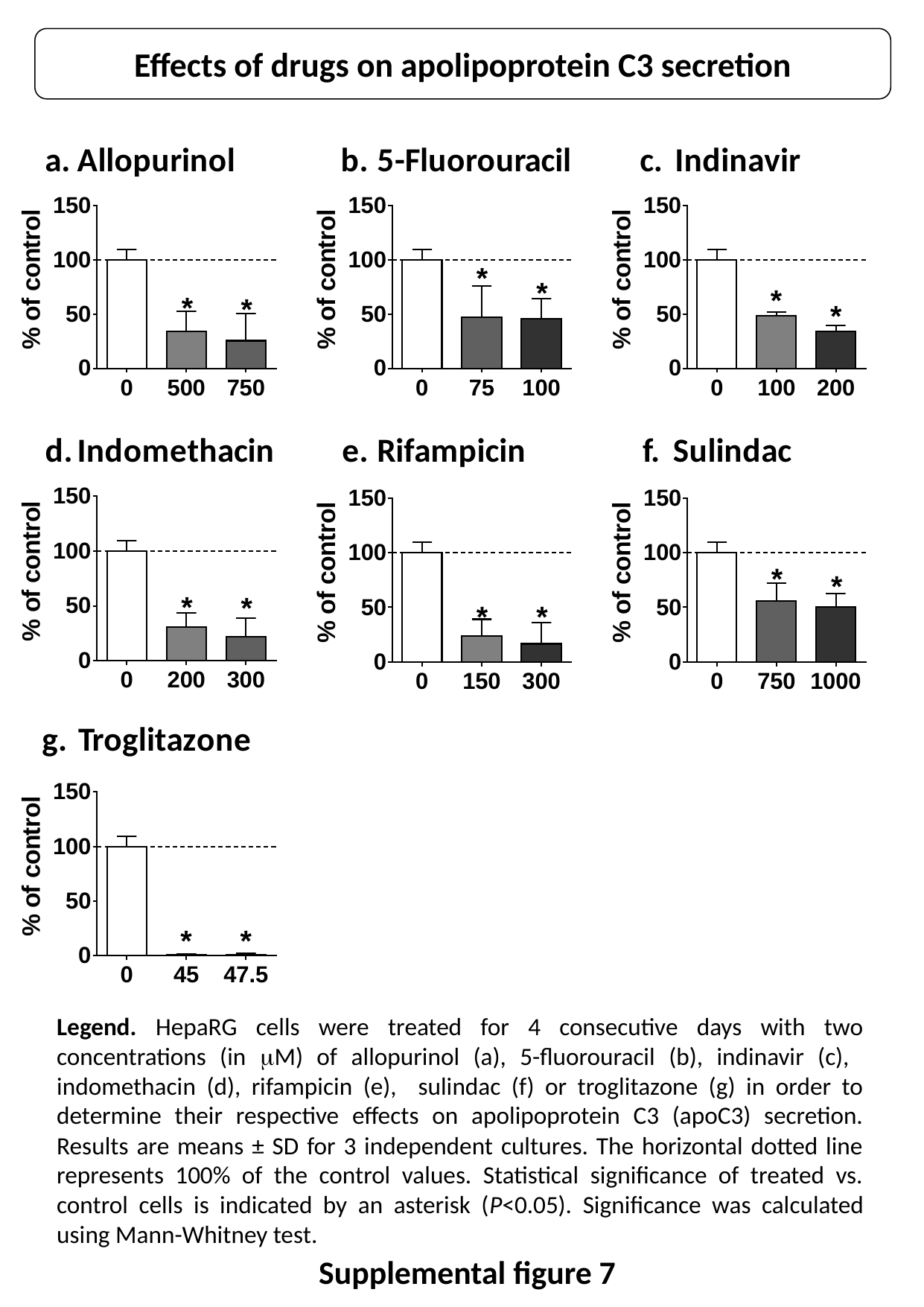

Effects of drugs on apolipoprotein C3 secretion
Legend. HepaRG cells were treated for 4 consecutive days with two concentrations (in mM) of allopurinol (a), 5-fluorouracil (b), indinavir (c), indomethacin (d), rifampicin (e), sulindac (f) or troglitazone (g) in order to determine their respective effects on apolipoprotein C3 (apoC3) secretion. Results are means ± SD for 3 independent cultures. The horizontal dotted line represents 100% of the control values. Statistical significance of treated vs. control cells is indicated by an asterisk (P<0.05). Significance was calculated using Mann-Whitney test.
Supplemental figure 7

## Slide 9
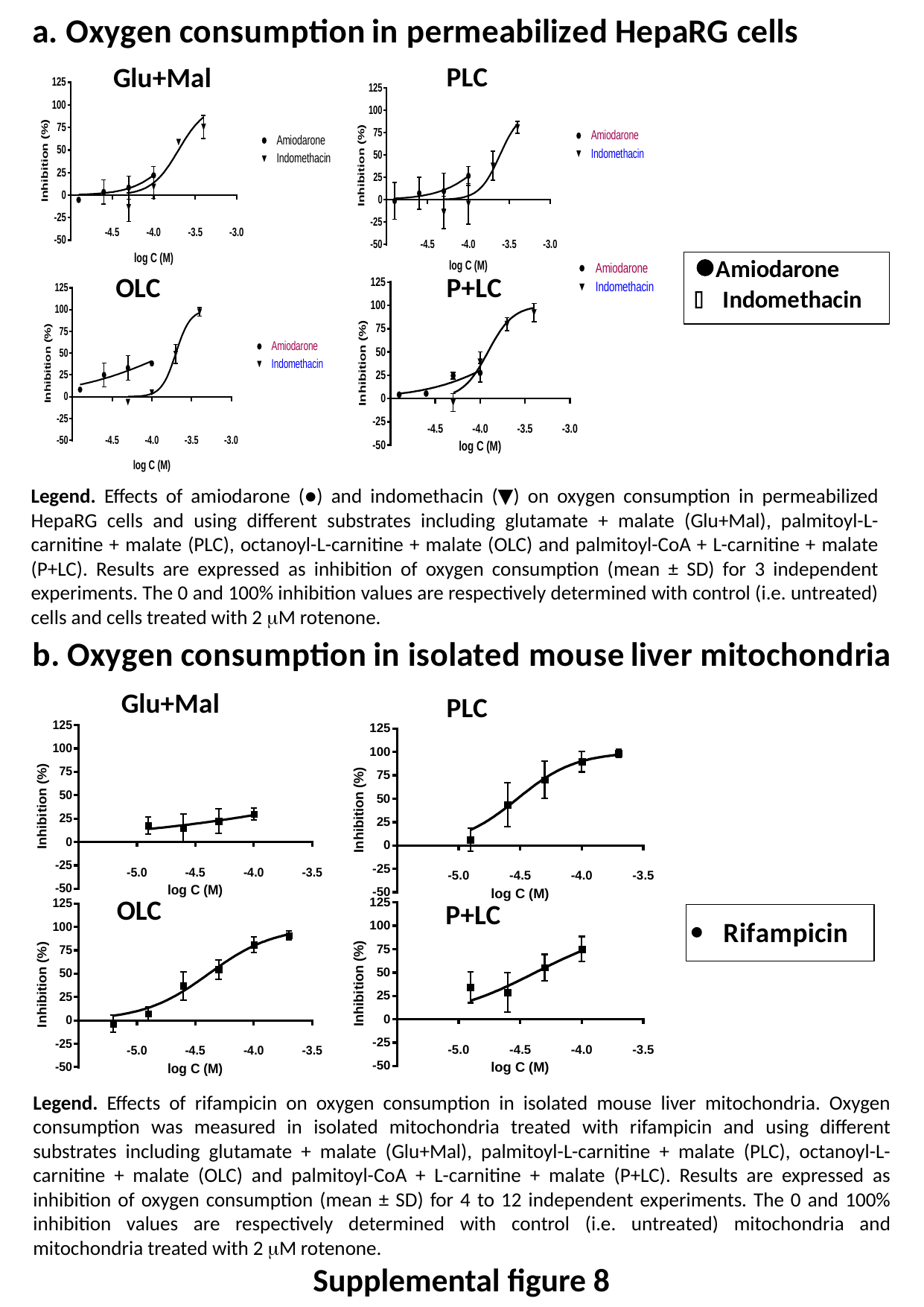

Legend. Effects of amiodarone (●) and indomethacin (▼) on oxygen consumption in permeabilized HepaRG cells and using different substrates including glutamate + malate (Glu+Mal), palmitoyl-L-carnitine + malate (PLC), octanoyl-L-carnitine + malate (OLC) and palmitoyl-CoA + L-carnitine + malate (P+LC). Results are expressed as inhibition of oxygen consumption (mean ± SD) for 3 independent experiments. The 0 and 100% inhibition values are respectively determined with control (i.e. untreated) cells and cells treated with 2 mM rotenone.
Legend. Effects of rifampicin on oxygen consumption in isolated mouse liver mitochondria. Oxygen consumption was measured in isolated mitochondria treated with rifampicin and using different substrates including glutamate + malate (Glu+Mal), palmitoyl-L-carnitine + malate (PLC), octanoyl-L-carnitine + malate (OLC) and palmitoyl-CoA + L-carnitine + malate (P+LC). Results are expressed as inhibition of oxygen consumption (mean ± SD) for 4 to 12 independent experiments. The 0 and 100% inhibition values are respectively determined with control (i.e. untreated) mitochondria and mitochondria treated with 2 mM rotenone.
Supplemental figure 8

## Slide 10
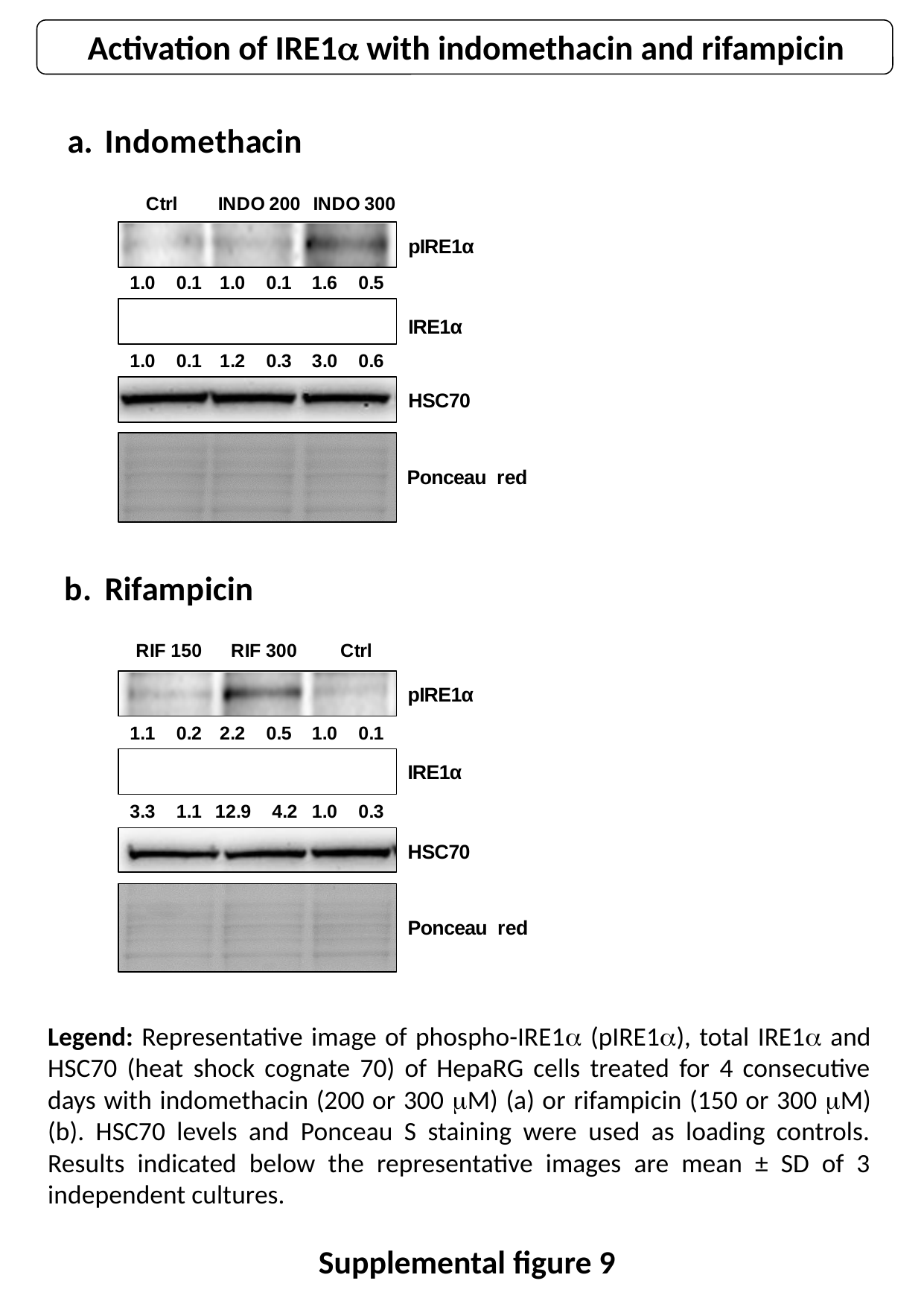

Activation of IRE1a with indomethacin and rifampicin
Legend: Representative image of phospho-IRE1a (pIRE1a), total IRE1a and HSC70 (heat shock cognate 70) of HepaRG cells treated for 4 consecutive days with indomethacin (200 or 300 mM) (a) or rifampicin (150 or 300 mM) (b). HSC70 levels and Ponceau S staining were used as loading controls. Results indicated below the representative images are mean ± SD of 3 independent cultures.
Supplemental figure 9

## Slide 11
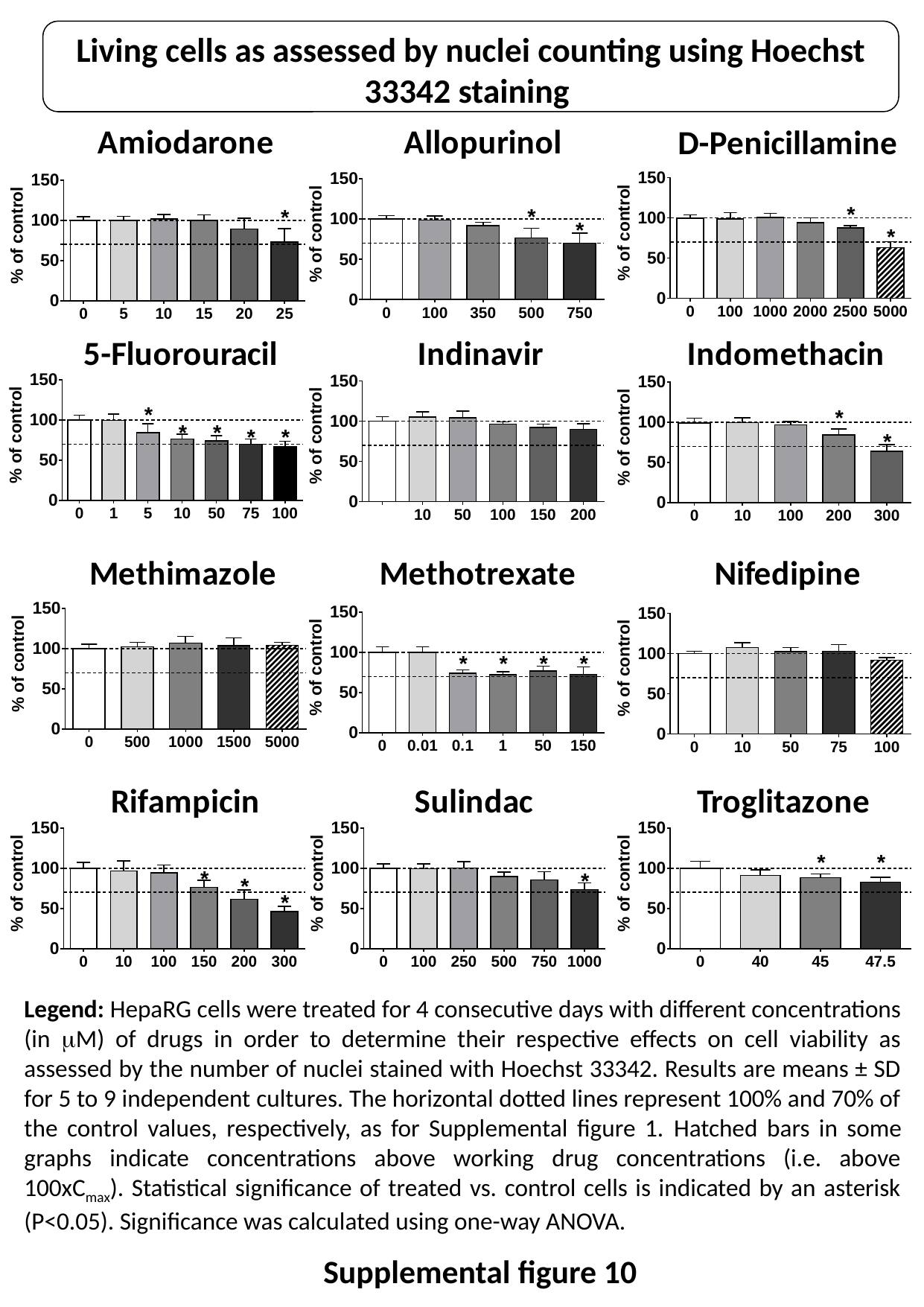

Living cells as assessed by nuclei counting using Hoechst 33342 staining
Legend: HepaRG cells were treated for 4 consecutive days with different concentrations (in mM) of drugs in order to determine their respective effects on cell viability as assessed by the number of nuclei stained with Hoechst 33342. Results are means ± SD for 5 to 9 independent cultures. The horizontal dotted lines represent 100% and 70% of the control values, respectively, as for Supplemental figure 1. Hatched bars in some graphs indicate concentrations above working drug concentrations (i.e. above 100xCmax). Statistical significance of treated vs. control cells is indicated by an asterisk (P<0.05). Significance was calculated using one-way ANOVA.
Supplemental figure 10

## Slide 12
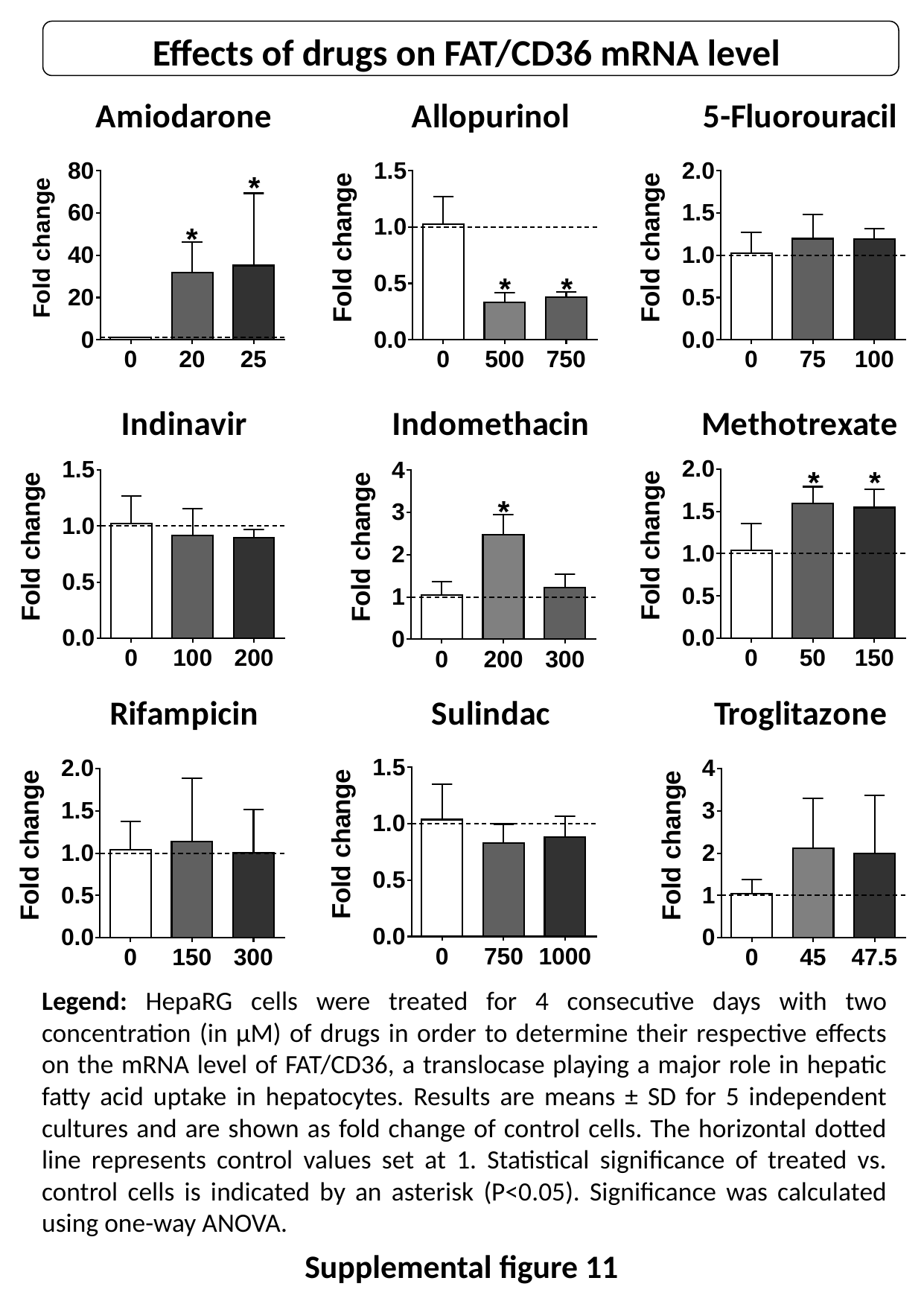

Effects of drugs on FAT/CD36 mRNA level
Legend: HepaRG cells were treated for 4 consecutive days with two concentration (in µM) of drugs in order to determine their respective effects on the mRNA level of FAT/CD36, a translocase playing a major role in hepatic fatty acid uptake in hepatocytes. Results are means ± SD for 5 independent cultures and are shown as fold change of control cells. The horizontal dotted line represents control values set at 1. Statistical significance of treated vs. control cells is indicated by an asterisk (P<0.05). Significance was calculated using one-way ANOVA.
Supplemental figure 11
