# Supplementary material for: Drug-induced hepatic steatosis in absence of severe mitochondrial dysfunction in HepaRG cells: proof of multiple mechanism-based toxicity
Source: Cell Biol Toxicol. 2020 Jun 14;37(2):151–75. doi: 10.1007/s10565-020-09537-1 (PMC8012331; doi:10.1007/s10565-020-09537-1)
Supplement: Supplementary file 2 — (DOCX 27 kb) [file 10565_2020_9537_MOESM2_ESM.docx]

**Supplementary material**

Drug-induced hepatic steatosis in absence of severe mitochondrial dysfunction in HepaRG cells: proof of multiple mechanism-based toxicity

Julien Allard, Simon Bucher, Julie Massart, Pierre-Jean Ferron, Dounia Le Guillou, Roxane Loyant, Yoann Daniel, Youenn Launay, Nelly Buron, Karima Begriche, Annie Borgne-Sanchez, Bernard Fromenty

**Supplemental Table 1** Presentation of the 12 steatogenic drugs selected in the study with their respective maximum plasma concentrations (C_max_) in patients

| **Drug**  **(abbreviation)** | **Therapeutic class and molecular weight (MW)** | **Maximum plasma concentration (C_max_) in patients and corresponding references** | **Concentration corresponding to 100xC_max_** | **Maximum concentration selected for the study** |
| --- | --- | --- | --- | --- |
|  |  |  |  |  |
| Amiodarone  (AMIO), positive control in this study | Antianginal, antiarrhythmic MW: 645 | Up to 1.87 mg/L (2.9 μM), Libersa CC et al., Br J Clin Pharmacol. 2000;49(4):373-8. | 290 μM | 25 μM (profound loss of cellular ATP for higher concentrations, as shown in Supp. fig 1) |
|  |  |  |  |  |
| Allopurinol  (ALLO) | Urate-lowering (antigout)  MW: 136 | Up to 2.05 mg/L (15 μM),  Tada H et al., J Clin Pharm Ther. 2003;28(3):229-34. | 1500 μM | 750 μM (loss of cellular ATP above 30% of the controls for higher concentrations, as shown in Supp. fig 1) |
|  |  |  |  |  |
| D-penicillamine  (DPEN) | Copper chelator MW: 149 | Up to 3.76 mg/L (25 μM),  Butler M et al., Arthritis Rheum. 1982;25(1):111-6. | 2500 μM | 2500 μM (slight loss of cellular ATP for this concentration, as shown in Supp. fig 1) |
|  |  |  |  |  |
| 5-Fluorouracil  (5FU) | Antineoplastic MW: 130 | Up to 55 mg/L (420 μM),  Casale F et al., Pharmacol Res. 2004;50(2):173-9. | 42000 μM | 100 μM (loss of cellular ATP above 30% of the controls for higher concentrations, as shown in Supp. fig 1) |
|  |  |  |  |  |
| Indinavir  (INDI) | Antiretroviral (anti-HIV)  MW: 614 | Up to 8 mg/L (13 μM),  Burger D et al., J Antimicrob Chemother. 2003;51(5):1231-8. | 1300 μM | 200 μM (not soluble above this concentration) |
|  |  |  |  |  |
| Indomethacin  (INDO) | NSAID  MW: 358 | Up to 8 mg/L (22 μM),  Alván G et al., Clin Pharmacol Ther. 1975;18(3):364-73. | 2200 μM | 300 μM (loss of cellular ATP above 30% of the controls for higher concentrations, as shown in Supp. fig 1) |
|  |  |  |  |  |
| Methimazole  (METHI) | Antithyroid  MW: 114 | Up to 1.5 mg/L (13 μM),  Cooper DS et al., J Clin Endocrinol Metab. 1984;58(3):473-9. | 1300 μM | 1500 μM (no loss of cellular ATP for this concentration, as shown in Supp. fig 1) |
|  |  |  |  |  |
| Methotrexate  (METHO) | Anti-rheumatoid, antipsoriatic, antineoplastic MW: 454 | Up to 594 μg/L (1.3 μM) for rheumatoid arthritis treatment,  Hoekstra M et al., J Rheumatol. 2004;31(4):645-8.  Up to 680 mg/L (1500 μM) for high dose chemotherapy, Holmboe L et al., Br J Clin Pharmacol. 2012;73(1):106-14. | 130 μM  150 mM | 150 μM (loss of cellular ATP below 30% of the controls for this concentration, as shown in Supp. fig 1) |
|  |  |  |  |  |
| Nifedipine  (NIF) | Antianginal, antihypertensive MW: 346 | Up to 250 μg/L (0.72 μM), Ahsan CH et al., Br J Clin Pharmacol. 1991;31(4):399-403. | 72 μM | 75 μM (no loss of cellular ATP for this concentration, as shown in Supp. fig 1) |
|  |  |  |  |  |
| Sulindac  (SUL) | NSAID  MW: 356 | Up to 11.4 mg/L (32 μM), Ravis WR et al., J Clin Pharmacol. 1993;33(6):527-34. | 3200 μM | 1000 μM (loss of cellular ATP above 30% of the controls for higher concentrations, as shown in Supp. fig 1) |
|  |  |  |  |  |
| Rifampicin  (RIF) | Antibiotic  MW: 823 | Up to 15.6 mg/L (19 μM), Ruslami R et al., Antimicrob Agents Chemother. 2007;51(7):2546-51. | 1900 μM | 300 μM (loss of cellular ATP above 30% of the controls for higher concentrations, as shown in Supp. fig 1) |
|  |  |  |  |  |
| Troglitazone  (TRO) | Antidiabetic  MW: 441 | Up to 2.83 mg/L (6.4 μM), Loi CM et al., Clin Pharmacokinet. 1999;37(2):91-104. | 640 μM | 47.5 μM (loss of cellular ATP above 30% of the controls for higher concentrations, as shown in Supp. fig 1) |
|  |  |  |  |  |

Abbreviations: NSAID, nonsteroidal anti-inflammatory drug; HIV, human immunodeficiency virus

**Supplemental Table 2** Presentation of the 12 steatogenic drugs selected in the study with their respective therapeutic blood (or plasma) concentrations in treated patients

| **Drug**  **(abbreviation)** | **Therapeutic class and molecular weight (MW)** | **Therapeutic blood (or plasma) concentration in mg/L (and µM) in patients according to Schulz M et al., Crit Care 2012 ;16(4):R136.** | **Maximum concentration selected for the study** |
| --- | --- | --- | --- |
|  |  |  |  |
| Amiodarone  (AMIO), positive control in this study | Antianginal, antiarrhythmic  MW: 645 | 1 - 2 mg/L (1.6 - 3.1 µM) | 25 μM |
|  |  |  |  |
| Allopurinol  (ALLO) | Urate-lowering (antigout)  MW: 136 | 2 - 19 mg/L (15 - 140 µM) | 750 μM |
|  |  |  |  |
| D-penicillamine  (DPEN) | Copper chelator  MW: 149 | 1.7 - 5.6 mg/L (11.4 - 37.6 µM) | 2500 μM |
|  |  |  |  |
| 5-Fluorouracil  (5FU) | Antineoplastic  MW: 130 | 0.05 - 0.3 mg/L (0.38 - 2.3 µM) | 100 μM |
|  |  |  |  |
| Indinavir  (INDI) | Antiretroviral (anti-HIV)  MW: 614 | > 0.1 mg/L (> 0.16 µM) | 200 μM |
|  |  |  |  |
| Indomethacin  (INDO) | NSAID  MW: 358 | 0.3 - 1 mg/L (0.83 - 2.8 µM) | 300 μM |
|  |  |  |  |
| Methimazole  (METHI) | Antithyroid  MW: 114 | 0.5 - 2.5 mg/L (4.4 - 22 µM) | 1500 μM |
|  |  |  |  |
| Methotrexate  (METHO) | Anti-rheumatoid, antipsoriatic, antineoplastic  MW: 454 | 0.04 mg/L (0.09 µM) | 150 μM |
|  |  |  |  |
| Nifedipine  (NIF) | Antianginal, antihypertensive  MW: 346 | 0.025 - 0.15 mg/L (0.07 - 0.43 µM) | 75 μM |
|  |  |  |  |
| Sulindac  (SUL) | NSAID  MW: 356 | 1 - 5 mg/L (2.8 - 14 µM) | 1000 μM |
|  |  |  |  |
| Rifampicin  (RIF) | Antibiotic  MW: 823 | 0.1 - 10 mg/L (0.12 - 12.2 µM) | 300 μM |
|  |  |  |  |
| Troglitazone  (TRO) | Antidiabetic  MW: 441 | 0.90 - 2.82 mg/L (2.0 - 6.4 µM)  Loi CM et al., J Clin Pharmacol 1999;39(9):920-6; Plosker GL and Faulds D. Drugs 1999;57(3):409‐438.  NB: data not available in Schulz M et al., Crit Care 2012; 16(4): R136. | 47.5 μM |
|  |  |  |  |

Abbreviations: NSAID, nonsteroidal anti-inflammatory drug; HIV, human immunodeficiency virus

**Supplemental Table 3** Primers used in the present study

| **Gene symbol (and alias)** | **Gene name** | **Accession number** | **Forward primer (5’-3’)** | **Reverse primer  (5’-3’)** |
| --- | --- | --- | --- | --- |
| *ACACA*  *(ACC1)* | Acetyl-CoA carboxylase alpha | NM_198839.2 | GGTGGATCGGAGATTTCATAGAG | AGGCTCCAGATGACGATAGA |
| *ACLY* | ATP citrate lyase | NM_198830.1 | GAGCAGCAGACCTATGACTATG | TGATGAGGATCTTGCCATCTG |
| *ANGPTL3* | Angiopoietin like 3 | NM_014495.4 | TACGCTACATCTAGTTGCGATTAC | CCACCAGCCTCCTGAATAAC |
| *APOB* | Apolipoprotein B | NM_000384.2 | CCCTCAGTCCTCTCCAGATAAA | GCTGCCTCTTCTTCCCAATTA |
| *APOC3* | Apolipoprotein C3 | NM_000040.3 | TCTGCCCGAGCTTCAGA | GCTGCTCAGTGCATCCTT |
| *ATF6* | Activating transcription factor 6 | NM_007348.4 | GTCAGAGAACCAGAGGCTTAAA | CCAACATGCTCATAGGTCCATA |
| *CD36* | CD36 molecule | NM_001001548.2 | GCCAGGTATTGCAGTTCTTTTC | TGTCTGGGTTTTCAACTGGAG |
| *DDIT3* (CHOP) | DNA damage inducible transcript 3 | NM_001195053.1 | GTCTAAGGCACTGAGCGTATC | CACTTCCTTCTTGAACACTCTCT |
| *ERN1* (IRE1α) | Endoplasmic reticulum to nucleus signaling 1 | NM_001433.5 | CCAGACAGACCTGCGTAAAT | CCGGTAGTGGTGCTTCTTATT |
| *FASN* | Fatty acid synthase | NM_004104.5 | CTCATCAAGTGGGACCACAG | GCTGGTGTCGATGTTGTAGAT |
| *GAPDH* | Glyceraldehyde-3-phosphate dehydrogenase | NM_001256799.2 | AGCCTCAAGATCATCAGCAAT | GTCATGAGTCCTTCCACGATAC |
| *HSPA5* (BIP) | Heat shock protein family A (Hsp70) member 5 | NM_005347.5 | TGGAGGTGGGCAAACAAA | AACTGCATGGGTAACCTTCTT |
| *MTTP* | Microsomal triglyceride transfer protein | NM_000253.3 | TACCAGGCTCATCAAGACAAAG | CTGACACCCAAGACCTGATTT |
| *P4HB* (PDI) | Prolyl 4-hydroxylase subunit beta | NM_000918.4 | TCGTGAACTGGCTGAAGAAG | GACTCCACGTCCTTGAAGAAG |
| *EIF2AK3* (PERK) | Eukaryotic translation initiation factor 2 alpha kinase 3 | NM_004836.7 | GACCTCAAGCCATCCAACATA | CTGGTCCATTGCAGTCACTAA |
| *SCD1* | Stearoyl-CoA desaturase | NM_005063.5 | AGTTCTACACCTGGCTTTGG | GTTGGCAATGATCAGAAAGAGC |
